# Policies and Problems of Modernizing Ethnomedicine in China: A Focus on the Yi and Dai Traditional Medicines of Yunnan Province

**DOI:** 10.1155/2020/1023297

**Published:** 2020-08-14

**Authors:** Zhiyong Li, Caifeng Li, Xiaobo Zhang, Shihuan Tang, Hongjun Yang, Xiuming Cui, Luqi Huang

**Affiliations:** ^1^Faculty of Life Science and Technology, Kunming University of Science and Technology, Kunming, Yunnan, China; ^2^School of Pharmacy, Minzu University of China, Beijing, China; ^3^Yunnan Province Resources of Development and Collaborative Innovation Center for New Traditional Chinese Medicine, Kunming, Yunnan, China; ^4^Jiangxi University of Traditional Chinese Medicine, Nanchang, Jiangxi, China; ^5^State Key Laboratory Breeding Base of Dao-Di Herbs, National Resource Center for Chinese Materia Medica, China Academy of Chinese Medical Sciences, Beijing, China; ^6^Institute of Chinese Materia Medica, China Academy of Chinese Medical Sciences, Beijing, China

## Abstract

Yunnan is a multiethnic province in southwest China, rich in Materia medica resources, and is popularly known as the kingdom of plants. Biomedicine and public health industry have been the industrial pillars of Yunnan since 2016, which is the important pharmaceutical industrial base for Dai and Yi medicine in China. This review of the Yunnan ethnic medicine industry describes some of the problems to be solved in the development of sustainable ethnomedicine in China. We investigated Chinese patent medicines (CPMs) declared as ethnomedicine on the drug instructions and identified 28 Dai patent medicines (DPMs) and 73 Yi patent medicines (YPMs) that were approved for clinical use in China. In further research, the clinical indications of these CPMs were determined, and the quality standard of medicinal materials and their usage frequencies in DPMs and YPMs were investigated. We also collected and analyzed the data on use of botanical and animal sources of medicines, the rare and endangered medicinal materials, and toxic medicines in DPMs and YPMs. The application of zootherapy in Yi traditional medicine was introduced from its abundant ancient documents and records; based on the “YaGei” theory in Dai traditional medicine, toxic medicines can be relatively safe in DPMs. However, for promoting the Yunnan traditional medicine industry, it is necessary to strengthen medical research to expand evidence-based clinical practice and balance ethnomedicine production and sustainable utilization of Materia medica resources, especially the animal sources of medicines, toxic medicines, and the protected wild resources reported in this survey. Only in this way can industrialization of ethnomedicine promote the improvement of human health.

## 1. Introduction

Evidence of the first human use of plants as medicines was observed in the fossil record of the Middle Paleolithic period, which began approximately 60,000 years ago [1]. Traditional medical knowledge and practices developed in different civilizations by the trial-and-error use of local botanicals and other biomaterial resources that accumulated slowly over long periods of time [2]. The World Health Organization (WHO) estimates that herbal medicines currently serve the health needs of approximately 80% of the world's population, especially millions of people living in the vast rural areas of developing countries [3]. The Chinese have one of the oldest and distinct medical systems in the world. Traditional Chinese Medicine (TCM) has a written history of nearly 3000 years and is widely practiced in China [4]. China is a multiracial country with 56 nationalities, 55 of which are officially recognized as ethnic minorities in 18 provinces of China. Each ethnic minority, e.g., the Tibetans, Mongols, Uygurs, Dai, Yi, and Miao, has its own traditional medicine, and each differs slightly in theory and practice from TCM. Ethnomedicine thus refers to the use of traditional medicine guided by the medical theory and practical experience of each ethnic minority [5]. Since 2017, the Law of the People's Republic of China on Traditional Chinese Medicine has given ethnomedicine a relatively independent status although it is considered as part of TCM. The importance of ethnomedicine has been increasing in China since 1951 as discussed in detail below. *Chinese Traditional Medicine Statistics* published in 2015 by the National Administration of Traditional Chinese Medicine of China and the *Investigation and Analysis of Quality Standards of Ethnomedicines in Nine Provinces of China* published in 2015 by the National Medical Products Administration of China listed 161 pharmaceutical enterprises that produced 4317 ethnic patent medicines (EPMs) and nearly 4000 in-hospital preparations of ethnomedicines used in 253 ethnic hospitals. A total of 39 EPMs were included in *Chinese Pharmacopoeia* (2015 edition). The fourth national survey of Chinese Materia medica resources is underway with the objective of determining the status of the available resources and investigating the modern value of herbal medicine including ethnic and folk medicines [6]. However, the national application of ethnic medicine in China is a complex issue that involves public policy, ethnic culture, livelihood status, regional economies, the protection of wild resources, etc.

Yunnan is a multiethnic province in southwest China. In addition to the Han nationality, there are 25 ethnic minorities with a population of more than 6,000, including the Yi, Hani, Bai, Dai, Zhuang, Miao, Hui, and Tibetan. The population of ethnic minorities is estimated at over 16 million, accounting for 33.4% of the provincial total population. The Dai and Yi traditional medicine are the representatives of ethnomedicine practiced in Yunnan. Tibetan medicine as practiced in Shangri-La will be described in a subsequent review. Yunnan Province is an important pharmaceutical industry center for Dai medicine and Yi medicine. For example, Yunnan Baiyao, a highly effective patent medicine has originated from the ancient Yi prescription [5]. Biomedicine and public health industry have been the major industries in Yunnan since 2016, and more than 2000 ethnic medicinal resources and more than 10,000 folk prescriptions are native to Yunnan [7]. This review focuses on the Yi and Dai traditional medicine in Yunnan and the potential problems to be encountered in the development of policies favorable to ethnomedicine development.

## 2. Historical Changes of Chinese Ethnomedicine Policies

Since 1949, the Chinese government has successively introduced many policies to support and protect the development of ethnomedicine (Table 1). The *Ethnic Minorities Health Work Plan of China* published in 1951 recommended that native doctors who used herbs to cure diseases should be united and supported to the greatest extent. *The Decision on Health Reform and Development* published in 1997 discussed how ethnomedicine should be mined, organized, summarized, and improved. *The Decision on Further Strengthening Rural Health Work*, published in 2002, promoted the development and organization of ethnomedicine resources and technologies in rural regions. For many years, ethnic folk doctors in rural areas provided care in very much the same way as barefoot doctors, a nickname for part-time paramedical workers in rural areas who were trained for simple diagnoses and treatments in the 1960s and 1970s. During that time, China's health services could not cover all areas of the country, and the need of ethnic and folk medical practices was tacitly accepted. At present, the Tibetan, Mongolian, Uygur, Dai, Kazakh, Korean, Zhuang, and Hui medicine have set up physician certification systems for special skill or knowledge to support ethnic folk doctors obtaining legal medical qualification through certain procedures. The *Outline of a Strategic Plan for Development of Traditional Chinese Medicine* (2016–2030) published by the State Council of China promotes the development of ethnomedicine.

The status of ethnomedicine in China experienced a cognitive change with the publication of the *Regulations of the People's Republic of China on Traditional Chinese Medicine* in 2003, which required that the administration of ethnomedicine should be implemented with compliance to the regulations that apply to TCM and established a close relationship between ethnomedicine and TCM. *The law of the People's Republic of China on Traditional Chinese Medicine* currently defines ethnomedicine as one part of TCM, sharing a history and development with TCM that conform to the united national culture of China. Yunnan Province has published policies and plans to regulate the development of its rich medicine resources in the autonomous prefectures of Xishuangbanna Dai, Chuxiong Yi, Diqing Tibet, Chuxiong Yi, and Xishuangbanna Dai between 2016 and 2030 [8].

## 3. Ethnic Hospitals and Pharmaceutical Enterprises in Yunnan

Yunnan Province has two Yi autonomous prefectures, including Chuxiong Yi and Honghe Hani Yi, and two Dai autonomous prefectures, including Xishuangbanna and Dehong Dai Jingpo. There are also 11 Yi autonomous counties and 7 Dai autonomous counties. The Yunnan Traditional Yi Medicine Hospital, the largest Yi medical hospital in China, is located in Chuxiong City, Chuxiong Yi Autonomous Prefecture. More than 10 counties have Yi medical hospitals or outpatient departments located in villages and towns of Yunnan. Each of the two Dai autonomous prefectures has a large traditional Dai medicine hospital, the hospital of Dai traditional medicine of Xishuangbanna Dai Autonomous Prefecture and the hospital of Dai traditional medicine of Dehong Dai Jingpo Autonomous Prefecture. There are at least 6 Dai hospitals in several counties.

A total of 42 corporations are licensed to produce Dai patent medicines (DPMs) and Yi patent medicines (YPMs) in China. Two corporations are located outside Yunnan Province. Twenty corporations are in Kunming City, nine corporations are in Chuxiong Yi Autonomous Prefecture, three corporations are in Yuxi City, and two corporations are in Dali Bai Autonomous Prefecture. Wenshan City, Zhaotong City, and Xishuangbanna Dai Autonomous Prefecture have one pharmaceutical manufacturer each. The companies include the Yunnan Baiyao Group, which is best known for producing Baibaodan, the original name of Yunnan Baiyao, which was invented by Qu Huanzhang (AD 1880–1938). It also produces more than 300 other patent medicines and 19 dosage forms including Shu Lie An Capsule, Qiancao Nao Tong Oral liquid, Gu Feng Ning Capsule, Shang Yi Aerosol, Tong Shu Capsule, and Zhong Tong Liniment. In 2014, Dihon Pharmaceutical Co. was purchased by Bayer, a large German pharmaceutical company, which was marked as a significant entry for Bayer into the TCM marketplace. Dihon produces Dan E Fu Kang Ointment, Gan Dan Qing Capsule, Yu Mai Kou Yan liquid, and Wei Fu Shu Capsule.

## 4. Clinical Indications of Yi and Dai Medicines

**Figure 1:**
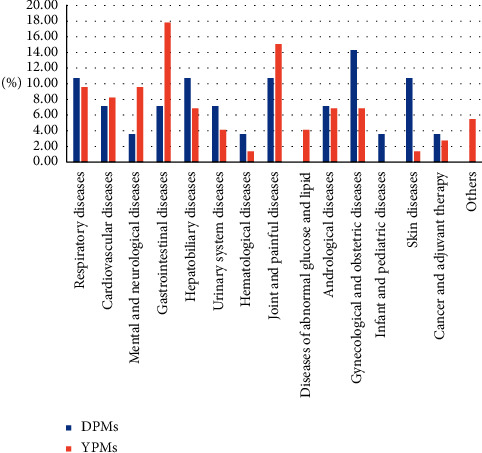
Clinical indications of DPMs and YPMs. DPMs, Dai patent medicines; YPMs, Yi patent medicines.

Based on the pharmaceutical instructions in which the properties of ethnic medicine were claimed, the clinical indications of DPMs and YPMs were surveyed and recorded, and 28 DPMs and 73 YPMs that could be approved for clinical use in China following the drug regulatory laws were identified. Fourteen DPMs such as Biao Re Qing Granule, Guan Tong Shu Oral liquid, and Hui Xue Sheng Capsule that have already been approved as over-the-counter (OTC) drugs accounted for 50% of the DPMs. The YPMs included 24 prescriptions such as Bai Bei Yi Fei Capsule, Chang Shu Zhi Xie Capsule, and Dan E Fu Kang Ointment, which have been approved as OTC drugs and account for 32.8% of the total YPMs. The information about these patent medicines is recorded in Tables S1 and S2. As shown in Figure 1, these DPMs and YPMs are used to treat respiratory, cardiovascular, mental, and neurological diseases among others. One example is Dan Deng Tong Nao Capsule (DDTN), of which *Erigeron breviscapus* (Vaniot) Hand. -Mazz (Dengzhanxixin), a component herb, has been recorded in the pattra-leaf scripture of Dai traditional medicine for 2500 years. DDTN when combined with rehabilitation training can improve the recovery of neurological function and the quality of life of stroke patients with cerebral infarction [9]. DDTN is also found to prevent cerebral injury in rats with middle cerebral artery-induced ischemic stroke by decreasing the intracellular Ca^2+^ concentration and inhibiting the release of excitatory amino acids [10].

## 5. Application of Quality Standards for Yi Medicine and Dai Medicine

In China, the quality standards of ethnic medicines and their patent medicines are based on the national standards included in the *Chinese Pharmacopoeia*, which has covered ethnomedicines since 1977. Previous research on EPMs in the *Chinese Pharmacopoeia* (2015 edition) found that some traditional medicines did not establish national quality standards, and that 71 traditional medicines, which include 39 EPMs, are not listed in the *Chinese Pharmacopoeia* [11]. This practice (called “upside-down standards,”) that involves quality standards for Chinese patent medicines (CPMs) but no standard for the composition of CPMs affects the safety of CPMs and the healthy development of the Chinese pharmaceutical industry.

**Figure 2:**
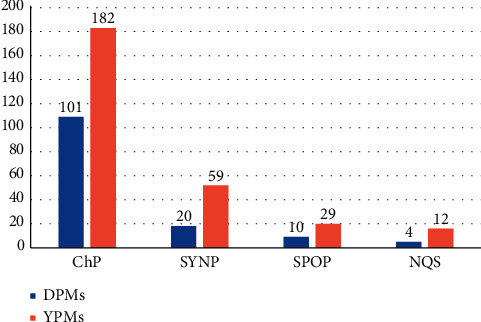
Quality standards of herbal medicines in DPMs and YPMs. ChP, *China Pharmacopoeia*; SYNP, Standards for Chinese medicinal materials in Yunnan Province; SPOP, Standards for Chinese medicinal materials in other province except Yunnan; NQS, No quality standard.

The provincial standards relating to the Tibetan, the Xinjiang Uygur, the Inner Mongolia, the Guangxi Zhuang Autonomous Region, Qinghai, Sichuan, and Yunnan and Guizhou provinces also apply to the regulation of the quality of ethnic medicines in China [12]. The academy group and enterprise standards are also applicable to the quality of ethnomedicine. The 28 DPMs identified in the survey include 101 traditional medicines with quality standards listed in the *Chinese Pharmacopoeia* (2015 edition). The quality standards of 30 traditional medicines are listed in the Standards for Chinese medicinal materials in Yunnan Province (SYNP) or other provincial quality standards (Figure 2). Four herbal medicines including *Aristolochia chuii* Wu (Dabaijie), *Michelia mahan* C. Y. Wu (Mahan), *Asparagus officinalis* L. (Xiaobaibu), and leaf and stem of *Vitex trifolia* L. do not have applicable quality standards or are out of date. The 73 YPMs identified in the survey included 182 traditional medicines with quality standards in the *Chinese Pharmacopoeia* (2015 edition). The quality standards of 88 traditional medicines are included in the SYNP or other provincial pharmacopoeias. Eleven herbal medicines have no applicable quality standards or are out of date, including the root of *Rosa odorata* (Andr.) Sweet var. gigantea (Crép.) Rhed. Et Wils. (Gugongguo), *Fibraurea recisa* Pierre (Dahuangteng), *Dolichos falcata* Klein (Damayao), *Cyanotis arachnoidea* G. B. Clarke (Lushuicao), *Bulbophyllum reptans* (Lindl.) Lindl. (Xiaolvji), *Adenophora bulleyana* Diels (Shashen), *Cynoglossum officinale* L. (Daotihu), *Crepis lignea* (Vant.) Babc. (Wanzhangsheng), *Cymbopogon distans* (Nees) Wats. (Yunxiangcao), and Ziheche and extract of *Hemsleya chinensis Cogn*. ex Forbes et Hemsl (Xuedan extract). More information is provided in Tables S3 and S4.

**Figure 3:**
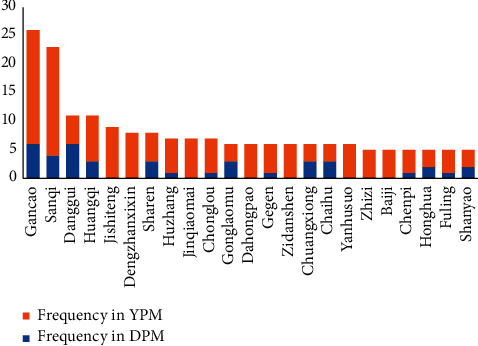
Usage frequencies of herbal medicines in DPMs and YPMs. DPM, Dai patent medicine; YPM, Yi patent medicine; Gancao, *Glycyrrhiza glabra* L. or *Hemsleya chinensis Cogn*.ex Forbes et Hemsl (Xuedan extract; Sanqi, *Panax notoginseng* (Burk.) F. H. Chen; Danggui, *Angelica sinensis* (Oliv.) Diels; Huangqi, *Astragalus propinquus* Schischkin; Jishiteng, *Paederia scandens* (Lour.) Merr.; Dengzhanxixin, *Erigeron breviscapus* (Vaniot) Hand. -Mazz; Sharen, *Amomum villosum* Lour.; Huzhang, *Polygonum cuspidatum* Sieb.et Zucc.; Jinqiaomai, *Fagopyrum dibotrys* (D. Don) Hara; Chonglou, *Paris polyphylla* Smith var. chinenisi (Franch) Hara; Gonglaomu, *Mahonia bealei* (Fort.) Carr.; Dahongpao, *Campylotropis hirtella* (Franchet) Schindler; Gegen, *Pueraria lobata* (Willd.) Ohwi; Zidanshen, *Salvia yunnanensis* C. H. Wright; Chuangxiong, *Ligusticum chuanxiong* Hort.; Chaihu, *Bupleurum scorzonerifolium* Willd; Yanhusuo, *Corydalis yanhusuo* W. T. Wang; Zhizi, *Gardenia jasminoides* Ellis; Baiji, *Bletilla striata* (Thunb.) Reichb. f.; Chenpi, *Citrus japonica* Blanco; Honghua, *Carthamus tinctorius* L.

The usage frequencies of traditional medicines in DPMs and YPMs are shown in Figure 3. *Glycyrrhiza uralensis* Fisch (Gancao), *Panax notoginseng* (Burk.) F. H. Chen (Sanqi), *Angelica sinensis* (Oliv.) Diels (Danggui), *Astragalus membranaceus* (Fisch.) Bge. Var. mongholicus (Bge.) Hsiao, and *Astragalus membranaceus* (Fisch.) Bge. (Huangqi) are the most frequently used genuine medicinal materials in Yunnan Province. The other medicines included in the SYNP as Dai medicines or Yi medicines are listed in Table 2.

## 6. Application of Medicinal Resources in Yi and Dai Medicine

### 6.1. Botanical, Animal, and Mineral Sources of Medicines Used in DPMs and YPMs

**Figure 4:**
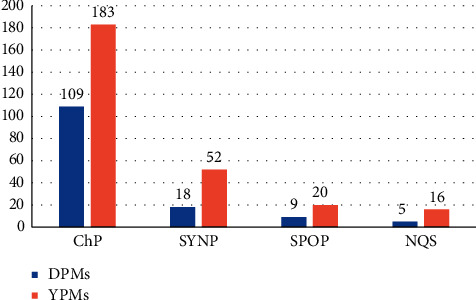
The use of botanical, animal, and mineral resources in DPMs and YPMs. DPMs, Dai patent medicines; YPMs, Yi patent medicines.

Differences in geographical and climatic conditions are reflected in distinct lifestyle, customs, cultures, and usage of medicinal resources by the residents of the various regions in China. In general, botanicals are the most widely used traditional medicines. DPMs and YPMs include 361 botanical medicines, 22 animal source of medicines, and 9 mineral medicines, as shown in Figure 4. YPMs contain more animal sources of medicines than DPMs. Yi people have a long history of hunting, and many ancient documents attest to the use of animal sources of medicines. The *Yi Nationality Offering Medicine Scriptures* (Yi Zu Xian Yao Jing), written in the early Qing Dynasty, notes that up to 92.8% of the prescriptions were animal sources of medicines and were divided into 12 types including insects, meat, bones, gallbladders, fat, blood, fish gall bladders, and hair. The *Book of Good medicines for Treating Diseases* (Yi Bing Hao Yao Shu, AD.1737) describes 152 animal sources of medicines that accounted for 35.7% of Yi medicines [13].

Animals are therapeutic arsenals with a significant role in healing. Zootherapy are derived from products of metabolism (e.g., corporal secretions and excrements) or from nonanimal materials such as nests or cocoons [14]. The reasonableness of zootherapy cannot be denied, and evidence supporting their use should be strengthened by modern scientific research. The animal sources of medicines in DPMs and YPMs are listed in Table 3. The use of some animal products in DPMs and YPMs is controversial, e.g., *Cordyceps sinensis*, *Moschus* deer musk, and bear bile, because these medicinal materials originate from protected wild animals or by harvesting activities that harm the ecological environment. Nevertheless, the production and use of such animal sources of medicines are restricted in China. China has joined and complied with many conventions on biological protection such as CITES (*Convention on International Trade in Endangered Species of Wild Fauna and Flora*) and the *Convention on Biological Diversity* and has implemented domestic animal protection regulations. However, the medicinal value of these and other animal sources of medicines cannot be ignored. Fortunately, substitutes are available because of the cultivation of *Cordyceps* and industrial production of artificial musk [15, 16]. Bile can be obtained from living, farmed bears but is ethically controversial [17]. Artificial bear bile has been reported to be effective on anticonvulsion, sedative, and choleretic [18].

### 6.2. Medicinal Parts of Botanical Medicines

**Figure 5:**
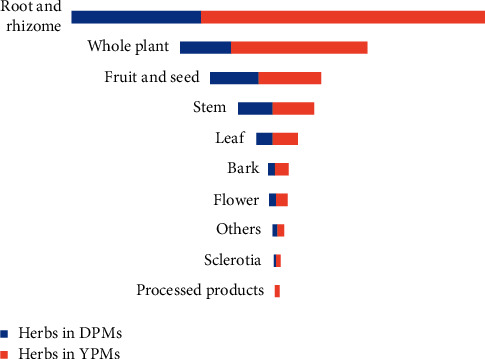
Relative contribution of the parts of medicinal plants to DPMs and YPMs.

Yunnan is rich in Chinese Materia medica resources and is known as the kingdom of plants. The plant parts used in herbal medicines include seeds, berries, roots, leaves, fruits, barks, and flowers and the whole plant itself. From ancient times to the present, people have used crude botanical materials as medicines to maintain vitality and cure disease [19]. The medicinal parts of botanical medicines in DPMs and YPMs are shown in Figure 5. The plant parts included in DPMs and YPMs are similar, with root and rhizomes, the whole plant, and fruit and seeds being the most frequent. The various parts of the medicinal plants contain active components that are responsible for their effectiveness [20] and physical properties that determine their names [21]. For example, Huangqin (*Scutellaria baicalensis* Georgi) is called “Rijishi” in the Yi language, in which “Ri” means herbaceous plant; “Ji” means root, the medicinal part of the plant; “Shi” indicates that the color is yellow [22]. The continuing usage of herbal medicines prepared from wild roots and rhizomes, fruits and seeds, and whole plants is not sustainable. The best strategy for balancing industrialization and resource protection is replacing wild with cultivated resources [23].

## 7. Rare and Endangered Medicinal Materials

The rapidly increasing demand for CPMs is likely to challenge the sustainability of herbal resources in China. At present, 80% of the most frequently used species cannot meet medical demand, and 1,800–2,100 medicinal species are facing extinction [24]. In the *China Plant Red Data Book* published in 1992, 388 plant species were listed as threatened, with 121 species as endangered and needing first-grade national protection, 110 species as rare needing second-grade national protection, and 157 species as vulnerable needing third-grade national protection. Of those plant species, 77 are herbal medicines that account for 19.86% of the threatened species [25]. The national key protection name list of wild animals in China includes 257 animal sources. The shortage of medicinal plants available to pharmaceutical companies can be partially reduced by the cultivation of at least 200 herbs, while some special herbs used in ethnomedicine are obtained by continuous wild collection without planned scientific cultivation. The rare medicinal materials used in DPMs and YPMs are listed in Table 4.

**Figure 6:**
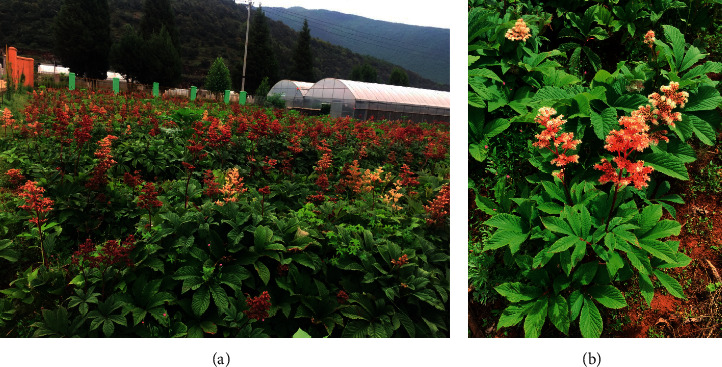
*Rodgersia sambucifolia* Hemsl. cultivated in the Meizi test ground (Lijiang, Yunnan). Toxic medicines used in DPMs and YPMs.

Those plants are protected by the Chinese government and some international nongovernment organizations such as the International Union for Conservation of Nature. *Cistanche deserticola* Y. C. Ma (Rouchongrong), *Panax ginseng* C. A. Mey (Renshen), *Glycyrrhiza aponic* Bat (Gancao), or other rare medicinal materials listed in the catalogs is protected and utilized sustainably in China. However, the number of endangered ethnic-specific medicines is far larger than that recorded in the catalogs; for example, more than 3000 tons of *Rodgersia sambucifolia* Hemsl. (Yantuo) are collected annually to produce YPMs. The availability of wild *Rodgersia* plants is sharply reduced, and resources are severely damaged in Luquan, Yongsheng, Yulong, Heqing, and Ninglang counties of Yunnan Province [26], while its cultivation research just began in recent years.  Figure 6 shows the planting situation of *Rodgersia sambucifolia* Hemsl. in the Meizi test ground, which is subordinate to the Institute of Alpine Economics and Botany, Yunnan Academy of Agricultural Sciences. Consequently, 30 traditional medicines have been listed in the *Rare Traditional Chinese Herbs of Yunnan Province in Urgent Needs* (RTCHYN), and those used in DPMs and YPMs are shown in Table 5 [27].

Medicines with pharmacological activities that are present in traditional ethnomedicine are likely to be clinically useful, but they may also be toxic, especially if used incorrectly or in the wrong amounts. Unlike that for modern drugs, the efficacy and toxicity assessments of these ethnomedicines are based on traditional knowledge and clinical experience rather than on laboratory evaluation [28]. Toxicity associated with the use of Chinese ethnomedicine may occur because of the environment, religious beliefs, or medical practices. The *Chinese Pharmacopoeia* includes 83 traditional medicines that are considered toxic [29]; some medicines listed in provincial standards of herbal medicines are also considered toxic [30].

Ten toxic medicines are used in 11 DPMs; six of those are included in the *Chinese Pharmacopoeia* (2015 edition) and four toxic medicines in the SYNP. One toxic medicine is used in Dai traditional medicine, and two toxic medicines are used in Yi traditional medicine. The 40 YPMs include 24 toxic herbs; 12 toxic medicines are in the *Chinese Pharmacopoeia* (2015 Edition) and 12 are in the SYNP. Four medicines are known as Yi medicines. Although some toxic ethnomedicines are used in DPMs and YPMs, the proprietary medicines are considered safe and approved for use in China because pharmaceutical processing, combining, and decocting contribute to reducing toxicity and enhancing efficacy. In the traditional Dai medicine (TDM), “YaGei” herbs are used to reduce toxicity. The “YaGei” detoxification theory is a unique supplement of TDM [31], and “YaGei” medicines are used as antidotes to relieve adverse reactions caused by food poisoning, drug poisoning, and other substances [32]. Dai people consume antidotes regularly to eliminate the microtoxins from the body, reduce the chance of illness, and prolong life.

Due to the lack of more pharmaceutical information disclosed, as well as the lack of basic research, the safety information of these DPMs and YPMs including toxic medicines is insufficient. The modern toxicological evidence of these toxic medicines is collected and summarized in Tables 6 and 7, focusing on Dai and Yi toxic medicines. The root of *Tripterygium hypoglaucum* (Levl.) Hutch (Huobahuagen) soaked in wine as an oral medicine is an ancient Yi medicine described in the *Ailao Materia Medica* in the treatment of arthritis, joint swelling, pain, bruises, and sprains [59]. *Boenning hauseniase silicarpa* Levl. (Shijiaocao) is described in the *Materia Medica in South Yunnan* (Dian Nan Ben Cao, AD.1396 -1476) written by Lan Mao as a bitter, pungent, and warm medicine used to treat chest pain, heartache, stomachache, and abdominal distension. Shijiaocao is also described in the *Ailao Materia Medica* as a treatment for sore throat, gastric pain, and dysentery. It is described as a cure of acute gastroenteritis in combination with the parasite of *Zanthoxylum bungeanum* in the *Wa Die Yi Medical Book*, which was written at the end of the Qing Dynasty [60]. Ancient documents describe the usage of the toxic herbs included in the medical practice of Yi, Dai, or other ethnic minorities in Yunnan. Modern toxicological study can ensure their safe and effective use, and further study is warranted to determine how the toxicity reduces while the prescription remains effective.

## 8. Concluding Remarks

Ethnomedicine is an important part of TCM that has a unique medical theoretical system and refers to a wide range of healthcare systems/structures, practices, beliefs, and therapeutic techniques that arise from indigenous cultural development. Thousands of years of ethnic amalgamation has produced diversity, integration, and differences among the traditional medicine of different Chinese ethnics. Approximately 8, 000 medicinal species are used by 40 ethnic minorities in China, which account for over 70% of the Chinese Materia medica resources. Data from the National Medical Products Administration of China show that there are more than 600 types of EPMs [12]. The *Chinese Pharmacopoeia* (1977 edition) began to cover DPMs, and some Miao patent medicines and YPMs were collected from the 2015 edition of *Chinese Pharmacopoeia*. Overall, a total of 39 CPMs were identified as EPMs, 26 EPMs as prescription drugs, and 13 EPMs as OTC drugs [11]. Prescriptions that are not approved by the government were not included in the review evaluated, but are still in use in clinics in regions of China inhabited by ethnic groups.

This review focuses on Dai and Yi traditional medicines in Yunnan Province because of their long histories and descriptions in the ancient medical literature. The earliest book of Yi traditional medicine that can be verified is the *Yuanyang Yi Medicine Book*, which was written in 957 AD and found in Yuanyang County of Yunnan Province in 1985 [14]. The earliest books of Dai traditional medicine that can be verified are *Ge Ya San Ha Ya*, which was written in 964-884 BC, and *Dang Ha Ya Long*, which was written in 1323 AD [61]. There are 1, 666 Dai medicines [62], nearly 1, 400 Yi medicines [13], and 400 medicines are listed in the *Yi Materia Medica* [63]. There are 478 Yi medical formulas described in *Chinese Yi Medicine Prescriptions Science* [64], and 200 Dai medical formulas are in *Study on Dai classical prescriptions of China* [65]. The numbers of folk formulas from Dai and Yi traditional medicine are not available to record yet. Just as the example of Yunnan Baiyao mentioned before, a series of ethnomedicines in Yunnan were successfully industrialized and modernized to promote the modern vitality of ancient ethnomedicines and thus serve a wide population range. The Tong Shu Capsule is a YPM produced by the Yunnan Baiyao Group that has been approved recently for phase II clinical research in the United States. Yunnan Province expects to produce TCMs including ethnomedicines with a value of 140 billion RMB in 2020 and an average annual growth of more than 15%, accounting for 75% of its production [65].

Five key conclusions can be drawn from this investigation of Dai and Yi medicines.

First, except for the Yunnan Baiyao Group and the Dihon Pharmaceutical Company, most of the pharmaceutical manufacturers of EPMs in Yunnan Province are small enterprises, thereby limiting research and development capacity. A search on the *China National Knowledge Infrastructure* (CNKI, http://www.cnki.net) found 163 articles that reported investigations of these 28 DPMs reviewed here and 59 articles about Yunnan Baiyao Aerosol, whereas it is only one of the CPMs produced by the Yunnan Baiyao Group. In 2015, 100 million bottles of Yunnan Baiyao Aerosol were produced, with a value of more 1.5 billion RMB. In the same year, the overall sales revenue of the Yunnan Baiyao Group was 20.74 billion RMB [7].

Second, the sale volumes of YPMs and DPMs cannot be grasped, and it is hard to determine the extent to which traditional medicines used in YPMs and DPMs are collected in the wild. This will be a challenge to sustainable utilization for Chinese Materia medica resources.

Third, the use of toxic medicine used in ethnomedicine is of concern. Herbal medicine containing aristolochic acid has been associated with nephropathy in Belgium [66], and the adverse events associated with the use of Xiao Chaihu Tang in Japan [67] have led to warnings of the safety of CPM. Scientific evidence is needed to demonstrate the rationale and necessity of using toxic herbs in EPMs.

Fourth, the identification and usage of traditional medicines vary among ethnic minorities because of differences in experiences of clinical practice. The survey of DPMs and YPMs showed the differences in the number of animal sources of medicines used by the Yi and Dai people. The differences were also found in the ancient medical literature of Yi and Dai minorities.

Fifth, Dai and Yi medical prescriptions were traditionally written in the Dai and Yi languages, but the current clinical indications of DPMs and YPMs are written in Chinese. Difficulties in translation have hampered evaluation of how these ethnic medicines are used. Efforts to obtain accurate translations will be the next important work.

The sale volumes of DPMs and YPMs are not available because they belong to trade secrets. Because the descriptions of ethnic medical prescriptions in the ancient literature were written in Yi and Dai languages, they are hard to comprehend. However, the medical practices and culture of ethnic minorities have existed in Yunnan for thousands of years and have resulted in written records of more than 1300 ethnic medicinal materials and nearly 30,000 folk prescriptions. The medical information has been passed on orally or via ancient documents written in various ethnic minority languages such as the *San Ma Tou Yi Medical Book* and the *Lao Wu Dou Yi Medical book* written in the late Qing Dynasty of China. The ongoing scientific investigation and sustainable utilization of medicine resources will help to increase the impact of ethnomedicines of Yunnan Province on improvement of human health.

## Figures and Tables

**Table 1:** History of Chinese ethnomedicine policy.

Time	Policy outline	Promulgator
1951	Ethnic minorities health work plan of China	Unknown
1984	Several opinions on strengthening ethnic minorities work	Ministry of Health and NEAC
1997	Decision on health reform and development	CPC Central Committee and the State Council
2002	Decision on further strengthening rural health work	CPC Central Committee and the State Council
2003	Regulations of the People's Republic of China on traditional Chinese medicine	The State Council
2007	Guiding opinions on strengthening the development of ethnomedicine	11 ministries including NATCM
2017	The law of the People's Republic of China on traditional Chinese medicine	NPC standing committee
2018	Some opinions on strengthening the ethnomedicine work in the new era	13 ministries including NATCM

NEAC: National Ethnic Affairs Commission of PRC (China); CPC Central Committee: the Central Committee of the Communist Party; NATCM: National Administration of Traditional Chinese Medicine of PRC (China); NPC: National People's Congress.

**Table 2:** Traditional medicines used in DPMs and YPMs and listed in the SYNP.

No	Scientific name	Pinyin name	MP	EM	Frequency
1	*Plumbago zeylanica* Linn.	Baihuadan	Stem and leaf	Yi	1
2	*Toddalia asiatica* (L.) Lam.	Feilongzhangxue	Stem	Yi	1
3	*Tripterygium hypoglaucum* (Levl.) Hutch	Huobahuagen	Root	Yi	3
4	*Inula cappa* (Buch -Ham) DC.	Yang'erju	Whole plant	Yi	2
5	*Geum aleppicum* Thumb. var. Chinese Bolle	Wuqihuanyangcao	Whole plant	Yi	3
6	*Rhodobryum giganteum* (Hook.) Par.	Huixincao	Whole plant	Yi	2
7	*Polygonum paleaceum* Wall. ex Hook.	Caoxuejie	Rhizome	Yi	2
8	*Polygala arillata* Buch. Ham. ex D. Dom	Jigen	Roots and rhizome	Yi	1
9	*Salvia yunnanensis* C. H. Wright	Zi Danshen	Root	Yi	6
10	*Ampelopsis delavayana* (Franch.) Planch.	Yuputao gen	Root	Yi	3
11	*Swertia patens* Burk.	Xiao'er futong cao	Whole plant	Yi	3
12	*Polygonum cuspidatum* Sieb.et Zucc.	Huzhangye	Leaf	Yi	1
13	*Cynodon dactylon* (L.) Pers.	Qianxiancao	Whole plant	Yi	1
14	*Potentilla fulgens* Wall. ex Hook	Guanzhong	Root	Yi	2
15	*Ainsliaea pertyoides* Franch. var. albo-tomentosa Beauv.	Yexiahua	Whole plant	Yi	1
16	*Valeriana jatamansi* Jones	Matixiang	Roots and rhizome	Yi	1
17	*Speranskia tuberculata* (Bunge) Baillon	Tougucao	Aerial part	Yi	3
18	*Arthromeris mairei* (Brause) Ching	Diwugong	Rhizome	Yi	1
19	*Schefflera venulosa* (Wight et Arn.) Harms	Qiyelian	Whole plant, stem, and leaf	Yi	3
20	*Boenninghausenia sessilicarpa* Levl.	Shijiaocao	Whole plant	Yi	2
21	*Oxalis corniculata* Linn.	Zajiacao	Whole plant	Yi	1
22	*Anemone rivularis* Buch. Ham. ex DC.	Huzhangcao	Root	Yi	2
23	*Opuntia stricta* (Haw.) Haw. var. dillenii (Ker Gawl.) Benson.	Xianrencao	Stem	Yi	1
24	*Dysosma versipellis* (Hance) M. Cheng ex Ying	Bajiaolian	Rhizome	Yi	4
25	*Jatropha curcas* L.	Gaotong	Root bark, stem bark	Yi	1
26	*Ficus tikoua* Bur.	Dibanteng	Cane	Yi	1
27	*Kadsura longipedunculata* Finet et Gagnep.	Wuxiangxueteng	Cane	Yi	3
28	*Leycesteria aponic* Wall. var. stenosepala Rehd.	Dazuifeng	Aerial part	Yi	1
29	*Anaphalis bulleyana* (J. F. Jeffr.) Chang	Wuxiangcao	Whole plant	Yi	1
30	*Craibiodendron yunnanense* W.W. Smith	Jinyezi	Leaf	Yi	1
31	*Phyllanthus urinaria* L.	Yexiazhu	Aerial part	Dai	2
32	*Brassica integrifolia* (West) O. E. Schulz ex Urb.	Kucaizi	Seed	Dai	1
33	*Zingiber purpureum* Rosc.	Zisejiang	Rhizome	Dai	1
34	*Polyrhachis dives* Smith	Weimayi	Body	Dai	4
35	*Phyllanthus niruri* L.	Zhuzicao	Whole plant	Dai	1
36	*Tacca chantrieri* Andre	Jiangenshu	Stem tuber	Dai	2
37	*Stephania epigaea* H. S. Lo	Diburong	Root tuber	Dai	1
38	*Streptocaulon juventas* (Lour.) Merr.	Tengkushen	Root	Dai	1
39	*Inula cappa* (Buch.-Ham) DC	Yangerjugen	Root	Dai	1
40	*Benincasa hispida* (Thunb.) Cogn.	Kudonggua	Fruit	Dai	1

DPMs, Dai patent medicines; YPMs, Yi patent medicines; SYNP, Standards for Chinese medicinal materials in Yunnan Province; MP, medicinal parts; EM, ethnic medicine.

**Table 3:** Medicinal sources from the animals used in DPMs and YPMs that are listed in the SYNP.

Scientific name	Pinyin name	MP	Standard	DPM	YPM
*Cervus nip port* Temminck	Lurong	Antler	ChP	1	0
*Cryptotympana pustulata* Fabricius	Chantui	Slough	ChP	2	0
*Gallus gallus domesticus* Brisson	Jineijing	Gizzard	ChP	1	1
*Polyrhachis dives* Smith	Heimayi	Body	SYNP	1	4
*Gekko gecko* Linnaeus	Gejie	Body	ChP	0	4
*Pheretima aspergillum* (E. Perrier)	Dilong	Body	ChP	0	4
*Bufo bufo gargarizans* Cantor	Chansu	Secretion	ChP	0	1
*Aspongopus chinensis* Dallas	Jiuxiangchong	Body	ChP	0	2
Selenarctos thibetanus Cuvier	Xiongdanfen	Bile	SYNP	0	1
*Bombyx mori* Linnaeus	Jiangchan	Body	ChP	0	1
*Periplaneta japonica* Linnaeus	Feilie	Body	SYNP	0	1
*Sepiella maindroni de* Rochebrune	Haipiaoqiao	Shell	ChP	0	1
*Moschus berezovskii* Flerov	Shexiang	Secretion	ChP	0	1
*Armadillidium vulgare* Latreille	Shufuchong	Body	SSDP	0	2
*Cervus nip port* Temminck	Lujiaoshuang	Antler colloid	ChP	1	1
*Cordyceps sinensis* (BerK.) Sacc.	Dongchongxiacao	Bacterial and insect complex	ChP	2	2

EPM, ethnic patent medicine; MP, medicinal parts; ChP, *Chinese Pharmacopoeia*; SSDP, Standards for Chinese medicinal materials in Shandong Province (2012).

**Table 4:** Rare medicinal materials used in DPMs and YPMs.

Herbal name	Scientific name	NPWP	IUCN	Proprietary	NPWM	UF
Gancao	*Glycyrrhiza uralensis* Fisch	II	LC	—	II	26
	*Glycyrrhiza inflata* Bat.	II	LC	—	II	
	*Glycyrrhiza glabra* L.	II	LC	—	II	
Renshen	Panax *ginseng* C. A. Mey	I	CR	—	II	4
Lianqiao	*Forsythia suspense* (Thunb.) Vahl.	—	—	—	III	4
Huangqin	*Scutellaria baicalensis* Georgi	—	—	—	III	4
Wuweizi	*Schisandra chinensis* (Turcz.) Baill.	II	LC	—	III	1
Lurong	*Cervus nip port* Temminck	—	—	—	I	1
	*Cervus elaphus* Linnaeus	—	—	—	I	
Rouchongrong	*Cistanche deserticola* Y. C. Ma	II	EN	—	III	1
	*Cistanche tubulosa* (Schenk) Wight	II	—	—	—	
Huangbai	*Phellodendron chinense* Schneid	—	—	—	II	2
Duzhong	*Eucommia ulmoides* Oliv.	—	—	—	II	1
Longdan	*Gentiana manshurica* Kitag.	—	—	—	III	3
	*Gentiana scabra* Bge	—	—	—	III	
	*Gentiana triflora* Pall.	—	—	—	III	
	*Gentiana regescens* Franch.	—	—	—	III	
Huanglian	*Coptis chinensis* Franch	—	—	Unique to China	II	1
	*Coptis deltoidea* C. Y. Cheng et Hsiao	—	VU	Unique to China	II	
	*Coptis teetoides* C. Y. Cheng.	—	—	—	II	
Houpu	*Magnolia officinalis* Rehd. et Wils	II	NT	Unique to China	II	1
	*Magnolia officinalis* Rehd. et Wils. var. biloba Rehd. et Wils	II	—	Unique to China	II	
Huangbai	*Phellodendron chinense* Schneid	—	—	—	II	2
Zicao	*Arnebia euchroma* (Royle) Johnst	—	—	—	III	1
Qingjiao	*Gentiana macrophylla* Pall.	—	—	—	III	1
	*Gentiana macrophylla* Maxim.	—	—	—	III	
	*Gentiana crassicaulis* Duthie ex Burk.	—	—	—	III	
	*Gentiana dahurica* Fisch	—	—	—	III	
Shexiang	*Moschus berezovskii* Flerov.	—	—	—	II	1
	*Moschus sifanicus* Przewalski.	—	—	—	II	
	*Moschus moschiferus* Linnaeus.	—	—	—	II	
Chonglou	*Paris polyphylla* Smith var. *chinensis* (Franch.) Hara	II	—	—	—	7

NPWP, National Key Protected Wild Plants of China (August 4, 1999); NPWM, National Key Protected Species of Wild Medicinal Materials of China (Dec. 1, 1987); IUCN, International Union for Conservation of Nature (CR, critically endangered; LC, least concern; EN, endangered; VU, vulnerable; NT, near threatened); UF, usage frequency in DPMs and YPMs.

**Table 5:** Traditional medicines listed in the RTCHYN.

Scientific name	Pinyin name	Medicinal parts	Regional distribution^*∗*^	Standard	UF
*Erigeron breviscapus* (Vaniot) Hand. -Mazz.	Dengzhanxixin	Whole plant	Areas except southwest of Yunnan	ChP	7
*Cordyceps sinensis* (BerK.) Sacc.	Dongchongxiacao	Bacterial and insect complex	Deqin, Shangri-La, Lijiang, Binchuan, Lvfeng, Guangtong	ChP	4
*Dracaena cochinchinensis* (Lour.) S. C. Chen A	Longxuejie	Resin	Jinping, Menglian, Pu'er, Jinghong, Zhenkang	SGZP	2
*Cyanotis arachnoidea* C. B. Clarke	Lushuicao	Whole plant	Menghai, Menglian, Jinghong, Jingdong, Mengzi, Anning, Kunming, Pingbian	No	1
*Swertia mileensis* T. N. He et W. L. Shi	Qingyedan	Whole plant	Mile	ChP	2
*Anisodus acutangulus* C. Y. Wu et C. Chen	Sanfensan	Roots	Lijiang	SYNP	1
*Hemsleya amabilis* Diels	Xuedan	Roots	Kunming, chongming, Binchuan, Eryuan, Dali, Heqing	No	1
*Bergenia purpurascens* (Hook.f.et Thoms.) Engl. var. delavayi (Franch.) Engl. et Irm.	Yanbaicai	Rhizome	Deqin, Weixi, Shangri-La, Lijiang, Dali, Qujing, Ludian, Zhaotong, Gongshan, Fugong	ChP	1

RTCHYN, *Rare Traditional Chinese Herbs of Yunnan Province in Urgent Needs*. ^*∗*^Regional distribution is from the *Flora of Yunnan* (*Science Press of China*, 2006). UF, usage frequency; ChP, *Chinese Pharmacopoeia*; SGZP, Standards for Chinese medicinal materials in Guizhou Province (2009); SYNP, Standards for Chinese medicinal materials in Yunnan Province (2005).

**Table 6:** Toxic medicines in DPMs.

Scientific name	Pinyin name	Toxicity degree	Standard	DPM	Modern toxicology	References
*Paris polyphylla* Smith var. *chinensis* (Franch) Hara	Chonglou	LT	ChP	RBQC	Toxic to the digestive system and has cardio toxicity and neurotoxicity, LD_50_ = 2.68 g/kg (mice, p.o.)	[33]
*Curculigo orchioides* Gaertn.	Xianmao	MT	ChP	LXBST	LD_50_ = 215.9 g/kg (ethanol extract, rats, p.o.) and damages the liver, kidney, and reproductive organs with oral administration of 120 g/kg (ethanol extract, rats, 6 months)	[34]
*Cnidium monnieri* (L.) Cuss.	Shechuangzi	LT	ChP	LXBST	Nausea and vomiting, decreased spontaneous activity, shortness of breath, unstable gait, and tremor (ethanol extract), LD_50_ = 17.45 g/kg (mice, p.o.), MTD = 1.50 g/kg, or LD_50_ = 3.45 g/kg (osthol, mice, p.o.)	[35–37]
*Zanthoxylum nitidum* (Roxb.) DC.	Liangmianzhen	LT	ChP	7-JDHXO	Nitidine chloride damages the liver and kidney cells and decreases the heart rate of zebrafish	[38]
*Pinellia ternate* (Thunb.) Breit.	Banxia	MT	ChP	SBZKG	LD_50_ = 42.7 ± 1.27 g/kg (mice, p.o.), damages the renal and liver, causes serious damage to gastric mucosa, and has significant toxicity on pregnancy maternal mice and embryo (total alkaloids)	[39]
*Prunus armeniaca* L. var. *ansu* Maxim	Kuxinren	LT	ChP	SBZKG	LD_50_ of amygdalin is 25 g/kg (mice, i.v.), 887 mg/kg (mice, p.o.) and hydrocyanic acid produced by amygdalin inhibits the activity of cytochrome oxidase, leading to cell respiration inhibition and cell death	[40]
*Plumbago zeylanica* Linn.	Bhuadan	LT	SYNP	DLBSC	Skin redness, swelling, and peeling when contacted and antiovulation activities for female rats (alcohol extract)	[41, 42]
*Tacca chantrieri* Andre	Jiangenshu	MT	SYNP	YGT	Diarrhea and vomiting in mild intoxication and intestinal mucosal exfoliation and hemorrhoea appear in severe poisoning patients	[43]
*Tripterygium hypoglaucum* (Levl.) Hutch	Huobahuagen	LT	SYNP	GTSL	LD_50_ = 79 g/kg (male mice, p.o.), LD_50_ = 100 g/kg (female mice, p.o.), and reversible antifertility effect	[44, 45]
*Erythrina variegata* L. var. orientalis (L.) Merr	Haitongpi	MT	SSCP	GTSL	Unknown	

HT, high toxicity; MT, medium toxicity; LT, low toxicity; SSCP, Standards for Chinese medicinal materials in Sichuan Province (2010); SYNP, Standards for Chinese medicinal materials in Yunnan Province (2005); RBQC, Ru Bi Qing Capsule; LXBST, Lu Xian Bu Shen Tablet; 7-JDHXO, 7-Jie Du Huo Xue Ointment; SBZKG, Shen Bei Zhi Ke Granular; DLBSC, Dan Lv Bu Shen Capsule; YGT, YaGei Tablet; GTSL, Guan Tong Shu Oral liquid.

**Table 7:** Toxic medicines in YPMs.

Scientific name	Pinyin name	Toxicity degree	Standard	YPM	Modern toxicology	References
*Paris polyphylla* Smith var. *chinensis* (Franch) Hara	Chonglou	LT	ChP	GFNC, NQSG, SYA, TSC, ZTL	—	—
*Osmunda japonica* Thunb.	Ziqiguanzhong	LT	ChP	SWYA	Unknown	—
*Evodia rutaecarpa* (Juss.) Benth.	Wuzhuyu	LT	ChP	GDQC, HWYP	LD_50_ is 2.70 mL/kg (volatile oil, mice, p.o.), one of the main target organ is the liver	[46]
*Bufo bufo gargarizans* cantor	Chansu	MT	ChP	CLTC	Ventricular arrhythmias and increasing the levels of Ca^2+^, CK, and LDH in the heart	[47]
*Artemisia argyi* Levl. Et Vant.	Aiye	LT	ChP	KSG	LD_50_ is 80.2 g/kg (aqueous extract, mice, p.o.), LD_50_ is 1.67 mL/kg (volatile oil, mice, p.o.), MTD is 75.6 g/kg (ethanol extract, mice, p.o.)	[48]
*Aconitum kusnezoffii* Reichb.	Caowu	HT	ChP	TXT	Causing serious cardiac dysfunction and damaging the nervous system. LD_50_ is 1.8 mg/kg, (aconitine, mice, p.o.), LD_50_ is 5.8 mg/kg (hypaconitine, mice, p.o.), and LD_50_ is 1.9 mg/kg (mesaconitine, mice, p.o.).	[49, 50]
*Papaver somniferum* L.	Yingsuqiao	MT	ChP	KLT	Main toxic components are morphine and codeine. Morphine with 60 mg causes poisoning and 250 mg leads to death	[51]
*Arisaema erubescens* (Wall.) Schott.	Tiannanxing	MT	ChP	TXT	Producing folate deficiency and injury to the kidneys	[52]
*Laggera pterodonta* (DC.) Benth.	Choulingdan	MT	ChP	LL, SKCG	LD_50_ is 1.19 g/kg (water extract, mice, i.p.)	[53]
*Prunus armeniaca* L. var. *ansu* Maxim	Kuxinren	LT	ChP	SKCG, CLTC	—	—
*Pinellia ternate* (Thunb.) Breit	Banxia	MT	ChP	WFSC, ZXASG	—	—
*Psammosilene tunicoides* W. C. Wu et C. Y. Wu	Jintiesuo	LT	ChP	ZTL	LD_50_ is 4.8471 (mice, p.o.) and toxic target organs include the lungs, spleen, and stomach	[54]
*Boenninghausenia sessilicarpa* Levl.	Shijiaocao	LT	SYNP	SAC, SKCG	The ether extract reduces the activity in mice by intraperitoneal injection	[33]
*Dysosma versipellis* (Hance) M. Cheng ex Ying	Bajiaolian	LT	SYNP	ZTL, HJXJC, SLAC, WJHXZTT	LD_50_ is 0.493 ± 0.032 g/kg (mice, p.o.) and is toxic to the heart and central nervous system, appearing excited then inhibited	[55]
*Millettia bonatiana* Pamp.	Dafahan	MT	SYNP	HSTT	Damages the stomach	[33]
*Craibiodendron yunnanense* W. W. Smith	Jinyezi	HT	SYNP	ZTL	Unknown	
*Tripterygium hypoglaucum* (Levl.) Hutch	Huobahuagen	MT	SYNP	ZTL, GFNC	—	
*Anemone rivularis* Bunch. Ham. ex DC.	Wuzhangcao	LT	SYNP	TYGT, YSL	Unknown	
*Delphinium yunnanense* Franch.	Daotihu	MT	SGZP	WHXZTC	Unknown	
*Dioscorea bulbifera* L.	Huangyaozi	LT	SGDP	FFLC, FFLG	LD_50_ is 25.49 g/kg (mice, i.p.), LD_50_ is 79.98 g/kg, 250.3 g/kg, or 544 g/kg (mice, p.o.), toxic target organs including the liver and kidney	[56, 57]
*Clematis apiifolia* var. *argentilucida* (H. Leveille & Vaniot) W. T. Wang	Shanmutong	LT	SHNP	NQSG	Unknown	
*Anisodus acutangulus* C. Y. Wu et C. Chen	Sanfensan	HT	SYNP	TXT	Unknown	
*Datura stramonium* L.	Mantuoluoye	MT	SYNP	YWNC	Shortness of breath and death after nerve stimulation	[33]
*Aconitum brachypodum* Diels.	Xueshangyizhihao	HT	SHNP	ZTL	LD_50_ are 6766.928 and 5492.337 mg/kg (petroleum ether extracts, N-butanol extracts, mice, p.o.)	[58]

HT, high toxicity; MT, medium toxicity; LT, low toxicity. ^*∗*^The herb has more than two origins of species, only one origin is shown. SGZP, Standards for Chinese medicinal materials in Guizhou Province (2009); SGDP, Standards for Chinese medicinal materials in Guangdong Province (2011); SHNP, Standards for Chinese medicinal materials in Hunan Province (2009); SYNP, Standards for Chinese medicinal materials in Yunnan Province (2005). GFNC, Gu Feng Ning Capsule; NQSG, Niao Qing Shu Granular; SYA, Shang Yi Aerosol; TSC, Tong Shu Capsule; ZTL, Zhong Tong Liniment; SWYA, Shu Wei Yao Alcohol; GDQC, Gan Dan Qing Capsule; HWYP, Huoxiang Wan Ying Powder; CLTC, Chuan Luo Tong Capsule; KSG, Kang Shen Granular; TXT, Tian Xiang Tincture; KLT, Ke Tan Oral liquid; LL, Lingdancao Oral liquid; SKCG, Shijiaocao Ke Chuan Granular; WFSC, Wei Fu Shu Capsule; ZXASG, Zhi Xuan An Shen Granular; SAC, Shen An Capsule; HJXJC, Hong Jin Xiao Jie Capsule; SLAC, Shu Lie An Capsule; WJHXZTT, Wu Jin Huo Xue Zhi Tong Tablet; HSTT, Huzhang Shang Tong Tincture; GFNC, Gu Feng Ning Capsule; TYGT, Tianhusui Yu Gan Tablet; YSL, Yan Shu Oral liquid; WHXZTC, Wujin Huo Xue Zhi Tong Capsule; FFLC, Fu Fang Luxiancao Capsule; FFLG, Fu Fang Luxiancao Granular; NQSG, Niao Qing Shu Granular; YWNC, Yun Wei Ning Capsule.

## Data Availability

The data used to support the findings of this study are available from the first author upon request.

## Abbreviations

CPM: Chinese patent medicine
ChP: *Chinese Pharmacopoeia*
CK: Creatine kinase
CR: Critically endangered
Ca^2+^: Calcium ion
DPM: Dai patent medicine
DDTN: Dan Deng Tong Nao Capsule
EM: Ethnic medicine
EN: Endangered
HT: High toxicity
IUCN: List of International Union for Conservation of Nature
LC: Least concern
LT: Low toxicity
LDH: Lactate dehydrogenase
LD50: Median lethal dose
i.v.: Intravenous
MP: Medicinal parts
MT: Medium toxicity
NPWP: National Key Protected Wild Plants of China (Aug 4, 1999)
NPWM: National Key Protected Species of Wild Medicinal Materials of China (December 1, 1987)
NQS: No quality standard
NT: Near threatened
OTC: Over-the-counter
p.o.: Per os
RTCHYN: Rare Traditional Chinese Herbs of Yunnan Province in Urgent Need
RMB: Renminbi
Ref.: Reference
SYNP: Standards for Chinese medicinal materials in Yunnan Province
SPOP: Standards for Chinese medicinal materials in other provinces
SSDP: Standards for Chinese medicinal materials in Shandong Province (2012)
SSCP: Standards for Chinese medicinal materials in Sichuan Province (2010)
SGZP: Standards for Chinese medicinal materials in Guizhou Province (2009)
TCM: Traditional Chinese medicine
UF: Usage frequency
VU: Vulnerable
WHO: World Health Organization
YPM: Yi patent medicine.

## Supplementary Materials

Supplementary Materials Tables S1 and S2: in this article, all the ethnic patent medicines (EPMs) produced in Yunnan Province were collected. The information of these EPMs is obtained from the National Medical Products Administration of China, and all EPMs listed in the article have been checked according to open drug instructions. Table S1 and Table S2 provide the following information including the drug name, Chinese name, approval number, clinical indications, dosage form, and prescription status of Yi patent medicines (YPMs) and Dai patent medicines (DPMs). Some of these data supported the results of “clinical indications of Yi and Dai medicines” and Figure 1 in this article. Tables S3 and S4: in this article, the investigation focused on the composition of each EPM, the types of medicinal resources and medicinal parts, and quality standards for medicinal materials used in EPMs, including botanical, animal, and mineral resources. All these data are listed as two tables, showing separately the composition information and quality standards in DPMs (Table S3) and YPMs (Table S4). The information includes the drug name and pinyin name, Chinese name, scientific name, and medicinal parts of medicinal materials used in EPMs, and also contains the quality standard information of medicinal materials which can be regarded as the most important key supporting data for this article.
