# Supplementary material for: Policies and Problems of Modernizing Ethnomedicine in China: A Focus on the Yi and Dai Traditional Medicines of Yunnan Province
Source: Evid Based Complement Alternat Med. 2020 Aug 14;2020:1023297. doi: 10.1155/2020/1023297 (PMC7443223; doi:10.1155/2020/1023297)
Supplement: Supplementary Materials — Tables S1 and S2: in this article, all the ethnic patent medicines (EPMs) produced in Yunnan Province were collected. The information of these EPMs is obtained from the National Medical Products Administration of China, and all EPMs listed in the article have been checked according to open drug instructions. Table S1 and Table S2 provide the following information including the drug name, Chinese name, approval number, clinical indications, dosage form, and prescription status of Yi patent medicines (YPMs) and Dai patent medicines (DPMs). Some of these data supported the results of “clinical indications of Yi and Dai medicines” and Figure 1 in this article. Tables S3 and S4: in this article, the investigation focused on the composition of each EPM, the types of medicinal resources and medicinal parts, and quality standards for medicinal materials used in EPMs, including botanical, animal, and mineral resources. All these data are listed as two tables, showing separately the composition information and quality standards in DPMs (Table S3) and YPMs (Table S4). The information includes the drug name and pinyin name, Chinese name, scientific name, and medicinal parts of medicinal materials used in EPMs, and also contains the quality standard information of medicinal materials which can be regarded as the most important key supporting data for this article. [file 1023297.f1.zip › other materials/table S1 and S2.docx]

**Table S1 Information of DPMs**

| No. | Drug Name | Chinese Name | Approval Number | Clinical Indications | Dosage form | OTC |
| --- | --- | --- | --- | --- | --- | --- |
| 1 | Biao Re Qing Granular (BRQG) | 表热清颗粒 | Z20026794 | Infection of the upper respiratory tract | Granular | Y |
| 2 | Dan Lv Bu Shen Capsule (DLBSC) | 丹绿补肾胶囊 | Z20025620 | Impotence and seminal emission | Capsule | N |
| 3 | Guan Tong Shu Oral liquid (GTSL) | 关通舒口服液 | Z20025408 | Joint pain and lumbar muscle strain | Oral liquid | Y |
| 4 | Hui Xin Kang Tablet (HXKT) | 回心康片 | Z20026037 | Coronary disease and hypertension | Tablet | N |
| 5 | Hui Xue Sheng Capsule (HXSC) | 惠血生胶囊 | Z20025066 | Anemia | Capsule | Y |
| 6 | San Yang Xue Dai Oral liquid (SYXDL) | 三阳血傣合剂 | Z20025065 | Anemia and leucopenia causing by tumor chemotherapy | Oral liquid | N |
| 7 | Lu Xian Bu Shen Tablet (LXBST) | 鹿仙补肾片 | Z20027604 | Impotence and weakness of waist and knee | Tablet | N |
| 8 | 7-Jie Du Huo Xue Ointment (7-JDHXO) | 七味解毒活血膏 | Z20026244 | Soft tissue injury and mild scald | Ointment | Y |
| 9 | Xiao Jie An Capsule (XJAC) | 消结安胶囊 | Z20025617 | Mammophilia, ovarian cyst and uterine leiomyoma | Capsule | N |
| 10 | Run Yi Rong Capsule (RYRC) | 润伊容胶囊 | Z20027531 | Acne | Capsule | Y |
| 11 | Shanzha Neijin Oral liquid (SNL) | 山楂内金口服液 | Z20027821 | Infantile malnutrition and indigestion | Oral liquid | Y |
| 12 | Shen Bei Zhi Ke Granular (SBZKG) | 参贝止咳颗粒 | Z20026126 | Chronic bronchitis | Granular | Y |
| 13 | Shen Cha Teabag (SCT) | 肾茶袋泡茶 | Z20026660 | Urinary tract infection | Teabag | Y |
| 14 | Shu Xin Tong Mai Capsule (SXTMC) | 舒心通脉胶囊 | Z20025429 | Coronary disease and angina pectoris | Capsule | N |
| 15 | Shuang Jiang Wei Tong Pills (SJWTP) | 双姜胃痛丸 | Z20026657 | Chronic superficial gastritis | Pills | Y |
| 16 | Xuan Ju Capsule (XJC) | 玄驹胶囊 | Z20026658 | Lumbar and knee pain | Capsule | Y |
| 17 | Xue Niao An Capsule (XNAC) | 血尿安胶囊 | Z20026104 | Urinary tract infection | Capsule | N |
| 18 | YaGei Tablet (YGT) | 雅解片 | Z20025088 | Gastric injury by alcohol and overeating | Tablet | Y |
| 19 | Ye Xia Zhu Tablet (YXZT) | 叶下珠片 | Z20026219 | Chronic hepatitis B and jaundice | Tablet | N |
| 20 | Ye Xia Zhu Capsule (YXZC) | 叶下珠胶囊 | Z20027597 | Chronic hepatitis B and jaundice | Capsule | N |
| 21 | Yi Kang Bu Yuan Granular (YKBYG) | 益康补元颗粒 | Z20026434 | Insomnia and amnesia | Granular | Y |
| 22 | Yi Shen Jian Gu Tablet (YSJGT) | 益肾健骨片 | Z20027061 | Chronic limb pain | Tablet | Y |
| 23 | Yin Qing Capsule (YQC) | 银芩胶囊 | Z20025199 | Infection of the upper respiratory tract | Capsule | Y |
| 24 | Zhuzi Gan Tai Capsule (ZGTC) | 珠子肝泰胶囊 | Z20026111 | Chronic hepatitis B | Capsule | N |
| 25 | Xiao Jie An Oral liquid (XJAL) | 消结安口服液 | Z20025884 | Mammophilia, ovarian cyst and uterine leiomyoma | Oral liquid | N |
| 26 | Huzhang Fanshi Liniment (HFL) | 虎杖矾石搽剂 | Z20025342 | Skin burns and scalds | Liniment | N |
| 27 | Ya Jiao Ha Dun Powder (YJHDP) | 雅叫哈顿散 | Z53021363 | Irregular menstruation and postpartum bleeding in women | Powder | N |
| 28 | Ru Bi Qing Capsule (RBQC) | 乳癖清胶囊 | Z20025068 | Mammary gland hyperplasia and menstrual breast pain | Capsule | N |

Note: Y (Yes) means that the Chinese patent drug belongs to OTC; N (No) means the Chinese patent drug not belongs to OTC.

**Table S2 Information of** **YPMs**

| No | Drug Name | Chinese Name | Approval Number | Clinical Indications | Dosage form | OTC |
| --- | --- | --- | --- | --- | --- | --- |
| 1 | Bai Bei Yi Fei Capsule (BBYFC) | 百贝益肺胶囊 | Z20025124 | Bronchitis and cough | Capsule | Y |
| 2 | Chang Shu Tablet (CST) | 肠舒片 | Z20025848 | Acute enteritis and dysentery | Tablet | N |
| 3 | Chang Shu Zhi Xie Capsule (CSZXC) | 肠舒止泻胶囊 | Z20025064 | Chronic diarrhea | Capsule | Y |
| 4 | Chang Wei Shu Capsule (CWSC) | 肠胃舒胶囊 | Z20026659 | Loss of appetite and abdominal pain | Capsule | N |
| 5 | Chuan Luo Tong Capsule (CLTC) | 喘络通胶囊 | Z20025126 | Bronchial asthma and emphysema | Capsule | N |
| 6 | Shu Lie An Capsule (SLAC) | 舒列安胶囊 | Z20025167 | Chronic prostatitis | Capsule | N |
| 7 | Dan Deng Tong Nao Capsule (DDTNC) | 丹灯通脑胶囊 | Z20026053 | Ischemic stroke | Capsule | N |
| 8 | Dan E Fu Kang Ointment (DEFKO) | 丹莪妇康煎膏 | Z20025253 | Female irregular menstruation, dysmenorrhea,  menstrual discomfort, and pelvic endometriosis | Ointment | Y |
| 9 | Danshen Yi Xin Capsule (DYXC) | 丹参益心胶囊 | Z20026028 | Coronary disease and angina pectoris | Capsule | N |
| 10 | Dan Wei Kang Capsule (DWKC) | 胆胃康胶囊 | Z20025134 | Jaundice, Bile Reflux Gastritis and Cholecystitis | Capsule | N |
| 11 | Deng Yin Nao Tong Capsule (DYNTC) | 灯银脑通胶囊 | Z20026228 | Cerebral ischemia | Capsule | N |
| 12 | E Qiu Qi Capsule (EQQC) | 饿求齐胶囊 | Z20025685 | Diarrhea | Capsule | Y |
| 13 | Fan Teng Zhi Injection (FTZI) | 矾藤痔注射液 | Z20026309 | Hemorrhoids | Injection | N |
| 14 | Fu Fang Dahongpao Zhi Xue Capsule  (FFDZXC) | 复方大红袍止血胶囊 | Z20025483 | Various hemorrhagic diseases, such as functional  uterine bleeding, bleeding after induced abortion,  epistaxis, gastric bleeding and hemorrhoids bleeding | Capsule | N |
| 15 | Fu Fang Luxiancao Granular (FFLG) | 复方鹿仙草颗粒 | Z20025653 | Primary hepatocellular carcinoma | Granular | N |
| 16 | Fu Fang Qinghao Spray (FFQS) | 复方青蒿喷雾剂 | Z20025887 | Hemorrhoids | Spray | Y |
| 17 | Fu Yi Shen Alcohol (FYSA) | 茯蚁参酒 | Z20026807 | Insomnia | Vinum | Y |
| 18 | Gan Dan Qing Capsule (GDQC) | 肝胆清胶囊 | Z20025161 | Cholecystitis and cholelithiasis | Capsule | N |
| 19 | Gu Feng Ning Capsule (GFNC) | 骨风宁胶囊 | Z20026229 | Rheumatoid arthritis and Ankylosing spondylitis | Capsule | N |
| 20 | He Wei Zhi Tong Capsule (HWZTC) | 和胃止痛胶囊 | B20020328 | Acute and chronic gastroenteritis, gastric and  duodenal ulcers, chronic colitis | Capsule | N |
| 21 | Wen Zhong He Wei Capsule (WZHWC) | 温中和胃胶囊 | Z20025689 | Chronic gastritis and duodenal ulcer | Capsule | N |
| 22 | Huzhang Shang Tong Tincture (HSTT) | 虎杖伤痛酊 | Z20025395 | Pain and swelling due to external injury | Tincture | Y |
| 23 | Hu Zhang Ye Capsule (HZYC) | 虎杖叶胶囊 | Z20026314 | Dizziness, dizziness and headache  caused by hypertension | Capsule | N |
| 24 | Huoxiang Wan Ying Powder (HWYP) | 藿香万应散 | Z20025180 | Gastrointestinal cold | Powder | Y |
| 25 | Jiang Zhi Tong Mai Capsule (JZTMC) | 降脂通脉胶囊 | Z20026429 | Hyperlipidemia | Capsule | N |
| 26 | Kang Shen Granular (KSG) | 康肾颗粒 | Z20025358 | Uremia | Granular | N |
| 27 | Ke Tan Oral liquid (KTL) | 咳痰合剂 | Z20025740 | Bronchitis or upper respiratory tract infection  appearing cough and phlegm | Oral liquid | N |
| 28 | Li Dan Jie Du Capsule (LDJDC) | 利胆解毒胶囊 | Z20025384 | Cholecystitis | Capsule | N |
| 29 | Lingdancao Oral liquid (LL) | 灵丹草合剂 | Z20026041 | Acute pharyngitis, tonsillitis and upper  respiratory tract infection | Oral liquid | Y |
| 30 | Long Jing Tong Lin Capsule (LJTLC) | 龙金通淋胶囊 | Z20025499 | Prostatitis, prostatic hyperplasia | Capsule | N |
| 31 | Lushuicao Capsule (LC) | 露水草胶囊 | Z20027532 | Type 2 diabetes | Capsule | N |
| 32 | Lvji Ke Chuan Granular (LKCG) | 绿及咳喘颗粒 | Z20025849 | Cough, night sweat | Granular | Y |
| 33 | Mitonghua Granular (MG) | 蜜桶花颗粒 | Z20027607 | Acute and chronic hepatitis | Granular | N |
| 34 | Niao Lu Kang Granular (NLKG) | 尿路康颗粒 | Z20027534 | non-gonococcal urethritis | Granular | N |
| 35 | Niao Qing Shu Granular (NQSG) | 尿清舒颗粒 | Z20026440 | chronic prostatitis | Granular | N |
| 36 | Ping Xuan Capsule (PXC) | 平眩胶囊 | Z20025826 | Somnipathy,dizziness, and palpitation | Capsule | N |
| 37 | Qiancao Nao Tong Oral liquid (QNTL) | 千草脑脉通合剂 | Z20025214 | Cerebral ischemia | Oral liquid | N |
| 38 | Qing Chang Tong Bian Capsule (QCTBC) | 清肠通便胶囊 | Z20025654 | Constipation | Capsule | Y |
| 39 | Rong Shuan Nao Tong Capsule (RSNTC) | 溶栓脑通胶囊 | Z20025006 | Cerebral ischemia | Capsule | N |
| 40 | She Chang Zhi Xie Powder (SCZXP) | 涩肠止泻散 | Z20025892 | Diarrhea | Powder | Y |
| 41 | Sha Mei Xiao Ke Capsule (SMXKC) | 沙梅消渴胶囊 | Z20025120 | Type 2 diabetes | Capsule | N |
| 42 | Shang Yi Aerosol (SYA) | 伤益气雾剂 | Z20026238 | Skin scald and injury | Aerosol | Y |
| 43 | Shen An Capsule (SAC) | 肾安胶囊 | Z20025529 | Lower urinary tract infection | Capsule | N |
| 44 | Shen Qi Xin Shu Capsule (SQXSC) | 参七心疏胶囊 | Z20025482 | Coronary disease and angina pectoris | Capsule | N |
| 45 | Shijiaocao Ke Chuan Granular (SKCG) | 石椒草咳喘颗粒 | Z20025635 | Chronic bronchitis | Granular | Y |
| 46 | Shu Mi Tong Capsule (SMTC) | 舒泌通胶囊 | Z20054802 | Hyperplasia of the prostate | Capsule | N |
| 47 | Shu Wei Yao Alcohol (SWYA) | 舒胃药酒 | Z20025389 | Indigestion | Vinum | Y |
| 48 | Tianhusui Yu Gan Tablet (TYGT) | 天胡荽愈肝片 | Z20025236 | Acute and chronic hepatitis | Tablet | N |
| 49 | Tian Xiang Tincture (TXT) | 天香酊 | Z20025711 | Soft tissue sprain and joint pain | Tincture | N |
| 50 | Tian Jing Yang Yan Capsule (TJYYC) | 调经养颜胶囊 | Z20025599 | Irregular menses and Dark skin in women | Capsule | Y |
| 51 | Tong Shu Capsule (TSC) | 痛舒胶囊 | Z20025478 | Traumatic pain and rheumatoid arthritis pain | Capsule | Y |
| 52 | Tong Shu Kou Shuang Capsule (TSKSC) | 通舒口爽胶囊 | Z20026241 | Constipation, gum swelling and pain | Capsule | Y |
| 53 | Wei Fu Shu Capsule (WFSC) | 胃复舒胶囊 | Z20025893 | Chronic superficial gastritis | Capsule | Y |
| 54 | Wen Ya Capsule (WYC) | 稳压胶囊 | Z20025645 | Hypertension | Capsule | N |
| 55 | Wujin Huo Xue Zhi Tong Capsule  (WHXZTC) | 乌金活血止痛胶囊 | Z20025249 | Various of pains, including limb  pain, rheumatic arthralgia and cancer pain | Capsule | N |
| 56 | Xiang Teng Capsule (XTC) | 香藤胶囊 | Z20025211 | Limb pain and rheumatic arthralgia | Capsule | N |
| 57 | Yanhu Wei An Capsule (YWAC) | 延胡胃安胶囊 | Z20026112 | Vomiting, stomachache, indigestion | Capsule | Y |
| 58 | Yan Lu Ru Kang Capsule (YLRKC) | 岩鹿乳康胶囊 | Z20025379 | Cyclomastopathy | Capsule | N |
| 59 | Yan Shu Oral liquid (YSL) | 咽舒合剂 | Z20025601 | Acute and chronic pharyngitis and tonsillitis | Oral liquid | N |
| 60 | Yi Xin Kang Capsule (YXKC) | 彝心康胶囊 | Z20025345 | Coronary diseases, ischemic  cerebrovascular disease | Capsule | N |
| 61 | Yu Mai Kou Yan Oral liquid (YMKYL) | 余麦口咽合剂 | Z20025158 | Mouth ulcer | Oral liquid | Y |
| 62 | Yun Wei Ning Capsule (YWNC) | 云胃宁胶囊 | Z20026811 | Gastric and duodenal ulcers, Chronic  gastritis and gastric spasm pain | Capsule | N |
| 63 | Zhi Xuan An Shen Granular (ZXASG) | 止眩安神颗粒 | Z20027533 | Vertigo, tinnitus, insomnia, palpitation | Granular | Y |
| 64 | Zhong Tong Liniment (ZTL) | 肿痛搽剂 | Z20026008 | Vertigo, tinnitus, insomnia, palpitation,  shoulder periarthritis, gout  arthritis, breast lobular hyperplasia. | Liniment | N |
| 65 | Zidan Huo Xue Tablet (ZHXT) | 紫丹活血片 | Z20025190 | Coronary heart disease, angina pectoris  and cerebral arteriosclerosis | Tablet | N |
| 66 | Zi Deng Capsule (ZDC) | 紫灯胶囊 | Z20025593 | Neck and shoulder pain caused by cervical spondylosis | Capsule | Y |
| 67 | Zi Jiao Xuan Tincture (ZJXT) | 紫椒癣酊 | Z20025684 | Tinea manus | Tincture | Y |
| 68 | Fu Fang Luxiancao Capsule (FFLC) | 复方鹿仙草胶囊 | Z20110028 | Primary hepatocellular carcinoma | Capsule | N |
| 69 | Hong Jin Xiao Jie Pill (HJXJP) | 红金消结浓缩丸 | Z20080315 | Female breast hyperplasia, uterine  leiomyoma, ovarian cyst | Pill | N |
| 70 | Hong Jin Xiao Jie Capsule (HJXJC) | 红金消结胶囊 | Z20026032 | Female breast hyperplasia, uterine  leiomyoma, ovarian cyst | Capsule | N |
| 71 | Shu Lie An Capsule (SLAC) | 舒列安胶囊 | Z20025167 | Chronic prostatitis | Capsule | N |
| 72 | Wu Jin Huo Xue Zhi Tong Tablet  (WJHXZTT) | 乌金活血止痛片 | Z20090688 | Various of pains, including limb pain,  rheumatic arthralgia and cancer pain | Tablet | N |
| 73 | Jin Wei Tai Capsule  (JWTC) | 金胃泰胶囊 | Z20026039 | Acute and chronic gastroenteritis, gastric  and duodenal ulcers, chronic colitis | Capsule | N |

Note: Y (Yes) means that the Chinese patent drug belongs to OTC; N (No) means the Chinese patent drug not belongs to OTC.
