# Supplementary material for: Policies and Problems of Modernizing Ethnomedicine in China: A Focus on the Yi and Dai Traditional Medicines of Yunnan Province
Source: Evid Based Complement Alternat Med. 2020 Aug 14;2020:1023297. doi: 10.1155/2020/1023297 (PMC7443223; doi:10.1155/2020/1023297)
Supplement: Supplementary Materials — Tables S1 and S2: in this article, all the ethnic patent medicines (EPMs) produced in Yunnan Province were collected. The information of these EPMs is obtained from the National Medical Products Administration of China, and all EPMs listed in the article have been checked according to open drug instructions. Table S1 and Table S2 provide the following information including the drug name, Chinese name, approval number, clinical indications, dosage form, and prescription status of Yi patent medicines (YPMs) and Dai patent medicines (DPMs). Some of these data supported the results of “clinical indications of Yi and Dai medicines” and Figure 1 in this article. Tables S3 and S4: in this article, the investigation focused on the composition of each EPM, the types of medicinal resources and medicinal parts, and quality standards for medicinal materials used in EPMs, including botanical, animal, and mineral resources. All these data are listed as two tables, showing separately the composition information and quality standards in DPMs (Table S3) and YPMs (Table S4). The information includes the drug name and pinyin name, Chinese name, scientific name, and medicinal parts of medicinal materials used in EPMs, and also contains the quality standard information of medicinal materials which can be regarded as the most important key supporting data for this article. [file 1023297.f1.zip › other materials/table S3.docx]

**Table S3 Composition information and Quality standards in DPMs**

| No. | EPMs Name | Pinying Name | Chinese Name | Scientific Name | Medicinal parts | Ref. |
| --- | --- | --- | --- | --- | --- | --- |
| 1 | Biao Re Qing Granular | Nanbanlangen | 南板蓝根 | *Baphicacanthus cusia* (Nees) Bremek | Rhizome and root | ChP |
|  |  | Shigao | 石膏 | CaSO_4_·2H_2_O | Mineral | ChP |
|  |  | Huangqin | 黄芩 | *Scutellaria baicalensis* Georgi | Root | ChP |
|  |  | Chaihu* | 柴胡 | *Bupleurum chinense* DC. | Root | ChP |
|  |  | Jinyinhua | 金银花 | *Lonicera japonica* Thunb. | Bud and opening flower | ChP |
|  |  | Gancao* | 甘草 | *Glycyrrhiza uralensis* Fisch. | Rhizome and root | ChP |
|  |  | Tuoshugen | 柘树根 | Unknown | - | No |
| 2 | Dan Lv Tonifying kidney Capsule | Hujiao | 胡椒 | *Piper nigrum* L. | Fruit | ChP |
|  |  | Ganjiang | 干姜 | *Zingiber officinale* Rosc. | Rhizome | ChP |
|  |  | Shegan | 射干 | *Belamcanda chinensis* (L.) DC. | Rhizome | ChP |
|  |  | Baihuadan | 白花丹 | *Plumbago zeylanica* Linn. | Stem and leaf | SYNP |
|  |  | Lvbaoteng | 绿包藤 | *Tinospora crispa* (Linnaeus) J. D.Hooker & Thomson | Cane | SHNP |
| 3 | Guan Tong Shu Oral liquid | Gonglaomu* | 功劳木 | *Mahonia bealei* (Fort.) Carr. | Stem | ChP |
|  |  | Xiqiancao* | 豨莶草 | *Siegesbeckia orientalis* L | Whole plant | ChP |
|  |  | Feilongzhangxue | 飞龙掌血 | *Toddalia asiatica* (L.) Lam. | Stem | SYNP |
|  |  | Xuemancao | 血满草 | *Sambucus adnata* Wall. | Whole plant | SYNP |
|  |  | Huobahuagen | 火把花根 | *Tripterygium hypoglaucum* (Levl.) Hutch | Root | SYNP |
|  |  | Haitongpi | 海桐皮 | *Kalopanax septemlobus* (Thunberg) Koidzum | - | - |
|  |  | Daokoucao | 倒扣草 | *Achyranthes aspera* Linnaeus | Whole plant | SHNP |
| 4 | Hui Xin Kang Tablet | Gouteng* | 钩藤 | *Uncaria rhynchphylla* (Miq.) Miq.ex | Stem and branch with hook | ChP |
|  |  | Heshouwu | 何首乌 | *Polygonum multiflorum* Thunb. | Root tuber | ChP |
|  |  | Huangqi* | 黄芪 | *Astragalus membranaceus* (Fisch.) Bge. Var. mongholicus (Bge.) Hsiao | Root | ChP |
|  |  | Sanqi | 三七 | *Panax notoginseng* (Burk.) F. H. Chen | Rhizome and root | ChP |
|  |  | Shanzha* | 山楂 | *Crataegus pinnatifida* Bge. | Fruit | ChP |
|  |  | Gancao* | 甘草 | *Glycyrrhiza uralensis* Fisch. | Rhizome and root | ChP |
|  |  | Huixincao | 回心草 | *Rhodobryum giganteum* (Hook.) Par. | Whole plant | SYNP |
| 5 | Hui Xue Sheng Capsule | Dangshen* | 党参 | *Codonopsis pilosula* (Franch.) Nannf. | Root | ChP |
|  |  | Danggui | 当归 | *Angelica sinensis* (Oliv.) Diels | Root | ChP |
|  |  | Shanyao | 山药 | *Dioscorea aponica* Thunb. | Rhizome | ChP |
|  |  | Dazao | 大枣 | *Ziziphus aponi* Mill. | Fruit | ChP |
|  |  | Baizhu | 白术 | *Atractylodes macrocephala* Koidz. | Rhizome | ChP |
|  |  | Longyanrou | 龙眼肉 | *Dimocarpus longan* Lour. | Aril | ChP |
|  |  | Huangqi* | 黄芪 | *Astragalus membranaceus* (Fisch.) Bge. Var. mongholicus (Bge.) Hsiao | Root | ChP |
|  |  | Sanqi | 三七 | *Panax notoginseng* (Burk.) F. H. Chen | Rhizome and root | ChP |
|  |  | Gancao* | 甘草 | *Glycyrrhiza uralensis* Fisch. | Rhizome and root | ChP |
|  |  | Sharen* | 砂仁 | *Amomum villosum* Lour. | Fruit | ChP |
|  |  | Dongchongxiacao | 冬虫夏草 | Cordyceps sinensis (BerK.) Sacc. | Fungus complex | ChP |
|  |  | Longxuejie | 龙血竭 | *Dracaena cochinchinensis* (Lour.) S. C. Chen A | Resin | SGZP |
| 6 | Jiang Jie Bu Xue Oral liquid | Shengjiang | 生姜 | *Zingiber officinale* Rosc. | Rhizome | ChP |
|  |  | Shanyao | 山药 | *Dioscorea aponica* Thunb. | Rhizome | ChP |
|  |  | Fuling | 茯苓 | *Poria cocos* (Schw.) Wolf | Sclerotia | ChP |
|  |  | Sharen* | 砂仁 | *Amomum villosum* Lour. | Fruit | ChP |
|  |  | Danggui | 当归 | *Angelica sinensis* (Oliv.) Diels | root | ChP |
|  |  | Sanqi | 三七 | *Panax notoginseng* (Burk.) F. H. Chen | Rhizome and root | ChP |
|  |  | Gancao* | 甘草 | *Glycyrrhiza aponic* Bat. | Rhizome and root | ChP |
|  |  | Longxuejie | 龙血竭 | *Dracaena cochinchinensis* (Lour.) S. C. Chen A | Resin | SGZP |
| 7 | Lu Xian Tonifying kidney Tablet | Lurong* | 鹿茸 | *Cervus aponi* Temminck | Antlers | ChP |
|  |  | Xianmao | 仙茅 | *Curculigo orchioides* Gaertn | Rhizome | ChP |
|  |  | Rouchongrong* | 肉苁蓉 | *Cistanche deserticola* Y.C.Ma | Succulent stems with scales | ChP |
|  |  | Jiucaizi | 韭菜子 | *Allium tuberosum Rottl*. ex Spreng | Seed | ChP |
|  |  | Renshen | 人参 | *Panax ginseng* C. A. Mey. | Rhizome and root | ChP |
|  |  | Buguzi | 补骨脂 | *Psoralea corylifolia* L. | Fruit | ChP |
|  |  | Danggui | 当归 | *Angelica sinensis* (Oliv.) Diels | Root | ChP |
|  |  | Tusizi* | 菟丝子 | *Cuscuta australis* R.Br. | Seed | ChP |
|  |  | Shechuangzi | 蛇床子 | *Cnidium monnieri* (L.) Cuss. | Fruit | ChP |
|  |  | Yinyanghuo * | 淫羊藿 | *Epimedium brevicornum* Maxim. | Leaf | ChP |
| 8 | 7-Jie Du Huo xue Ointment | Ercha | 儿茶 | *Acacia catechu* (L. f.) Willd. | Branch without bark | ChP |
|  |  | Yuxingcao | 鱼腥草 | *Houttuynia cordata* Thunb. | Whole plant | ChP |
|  |  | Mohanliang | 墨旱莲 | *Eclipta aponicas* L. | Whole plant | ChP |
|  |  | Liangmianzhen | 两面针 | *Zanthoxylum nitidum* (Roxb.) DC. | Root | ChP |
|  |  | Wuweizi* | 五倍子 | *Rhus potaninii* Maxim | Gall | ChP |
|  |  | Sumu | 苏木 | *Caesalpinia sappan* L. | Core material | ChP |
|  |  | Bohe | 薄荷脑 | DL-Menthol | - | ChP |
| 9 | Ru Bi An Xiao Capsule | Gonglaomu* | 功劳木 | *Mahonia bealei* (Fort.) Carr. | Stem | ChP |
|  |  | Yimucao | 益母草 | *Leonurus aponicas* Houtt. | Whole plant | ChP |
|  |  | Lianqiao | 连翘 | *Forsythia aponica* (Thunb.) Vahl | Fruit | ChP |
|  |  | Tufuling | 土茯苓 | *Smilax glabra* Roxb. | Rhizome | ChP |
|  |  | Jixueteng | 鸡血藤 | *Spatholobus suberectus* Dunn | Cane | ChP |
| 10 | Run Yi Rong Capsule | Pugongying* | 蒲公英 | *Taraxacum mongolicum* Hand. | Whole plant | ChP |
|  |  | Qianliguang | 千里光 | *Senecio scandens Buch*. -Ham. | Whole plant | ChP |
|  |  | Cebaiye | 侧柏叶 | *Platycladus orientalis* (L.) Franco | Branches and leaves | ChP |
|  |  | Daxueteng | 大血藤 | *Sargentodoxa cuneata* (Oliv.) Rehd. et Wils | Cane | ChP |
|  |  | Chaihu* | 柴胡 | *Bupleurum chinense* DC. | Root | ChP |
|  |  | Chuanmutong* | 川木通 | *Clematis armandi* Franch. | Cane | ChP |
|  |  | Baizhi* | 白芷 | *Angelica dahurica* (Fisch.ex Hoffm.) Benth. Et Hook.f. | Root | ChP |
|  |  | Zaojiaoci | 皂角刺 | *Gleditsia sinensis* Lam. | Thorn | ChP |
| 11 | Hawthorn and Chicken Neijin Oral liquid | Shanzha* | 山楂 | *Crataegus pinnatifida* Bge. | Fruit | ChP |
|  |  | Zangchangpu | 藏菖蒲 | *Acorus calamus* L． | Rhizome | ChP |
|  |  | Pipaye | 枇杷叶 | *Eriobotrya japonica* (Thunb.) Lindl | Leaf | ChP |
|  |  | Lianqiao | 连翘 | *Forsythia aponica* (Thunb.) Vahl | Fruit | ChP |
|  |  | Chantui | 蝉蜕 | *Cryptotympana pustulata* Fabricius | Cuticle | ChP |
|  |  | Jineijing | 鸡内金 | *Gallus gallus domesticus* Brisson | stomach | ChP |
|  |  | Jicai | 荠菜 | *Capsella bursapastoris* (L.) Medic | Whole plant | SHNP |
|  |  | Jishiteng | 鸡矢藤 | *Paederia scandens* (Lour.) Merr. | Whole plant | SHNP |
| 12 | Shen Bei Relieving cough Granular | Beishasheng | 北沙参 | *Glehnia littoralis* Fr.Schmidt ex Miq． | Root | ChP |
|  |  | Zhebeimu | 浙贝母 | *Fritillaria thunbergii* Miq | Bulb | ChP |
|  |  | Qianhu | 前胡 | *Peucedanum praeruptorum* Dunn | Root | ChP |
|  |  | Kuxinren* | 苦杏仁 | *Prunus armeniaca* L. var. ansu Maxim | Seed | ChP |
|  |  | Kuandonghua | 款冬花 | *Tussilago farfara* L. | Bud | ChP |
|  |  | Jingjie | 荆芥 | *Schizonepeta tenuifolia* Briq | Whole plant | ChP |
|  |  | Chenpi | 陈皮 | *Citrus aponicas* Blanco | Pericarp | ChP |
|  |  | Lianqiao | 连翘 | *Forsythia suspensa* (Thunb.) Vahl | Fruit | ChP |
|  |  | Chantui | 蝉蜕 | *Cryptotympana pustulata* Fabricius | Cuticle | ChP |
|  |  | Baibu* | 百部 | *Stemona sessilifolia* (Miq.) Miq. | Root tuber | ChP |
|  |  | Fabanxia | 法半夏 | *Pinellia aponic* (Thunb.) Breit | Root tuber | ChP |
| 13 | Shen Cha Teabag | Shencha | 肾茶 | *Clerodendranthus spicatus* (Thunberg) C. Y. Wu ex H. W. Li | Whole plant | SGZP |
| 14 | Shu Xin Tong Mai Capsule | Machixian | 马齿苋 | *Portulaca oleracea* L. | Whole plant | ChP |
|  |  | Qiannianjian | 千年健 | *Homalomena occulta* (Lour.) Schott | Rhizome | ChP |
|  |  | Chuanxiong | 川芎 | *Ligusticum chuanxiong* Hort. | Rhizome | ChP |
|  |  | Jiangxiang | 降香 | *Dalbergia odorifera* T. Chen | Core material | ChP |
|  |  | Danshen | 丹参 | *Scrophularia ningpoensis* Hemsl. | Rhizome and root | ChP |
| 15 | Shuang Jiang Relieving Stomachache Pills | Jianghuang | 姜黄 | *Curcuma longa* L. | Rhizome | ChP |
|  |  | Shichangpu | 石菖蒲 | *Acorus tatarinowii* Schott | Rhizome | ChP |
|  |  | Diburong | 地不容 | *Stephania epigaea* H. S. Lo | Root tuber | SYNP |
|  |  | Kucaizi | 苦菜子 | *Brassica integrifolia* (West). E. Schulz ex Urb. | Seed | SYNP |
|  |  | Zisejiang | 紫色姜 | *Zingiber purpureum* Rosc. | Rhizome | SYNP |
| 16 | Xuan Ju Capsule | Heimayi | 黑蚂蚁 | *Polyrhachis* dives Smith | Insect body | SYNP |
| 17 | Relieving Hematuria Capsule | Xiaoji | 小蓟 | *Cirsium setosum* (Willd.) MB. | Whole plant | ChP |
|  |  | Baimaogen | 白茅根 | *Imperata aponicas* Beauv. Var. major (Nees) C. E. Hubb. | Rhizome | ChP |
|  |  | Huangbai | 黄柏 | *Phellodendron chinese* Schneid. | Bark | ChP |
|  |  | Shencha | 肾茶 | *Clerodendranthus spicatus* (Thunberg) C. Y. Wu ex H. W. Li | Whole plant | SGZP |
| 18 | Antidoie Tablet (YaGei) | Gegen | 葛根 | *Pueraria lobata* (Willd.) Ohwi | Root | ChP |
|  |  | Gancao* | 甘草 | *Glycyrrhiza uralensis* Fisch. | Rhizome and root | ChP |
|  |  | Yangerju | 羊耳菊 | *Inula cappa* (Buch -Ham) DC. | Whole plant | SYNP |
|  |  | Jiangenshu | 箭根薯 | *Tacca chantrieri* Andre | Root tuber | SYNP |
|  |  | Dabaijie | 大百解 | Unknown | - | NO |
|  |  | Zhuyelan | 竹叶兰 | Unknown | - | NO |
| 19 | Ye Xia Zhu Tablet | Yexiazhu | 叶下珠 | *Phyllanthus urinaria* L. | Whole plant | SYNP |
| 20 | Ye Xia Zhu Capsule | Yexiazhu | 叶下珠 | *Phyllanthus urinaria* L. | Whole plant | SYNP |
| 21 | Yi Kang Bu Yuan Granular | Huangqi* | 黄芪 | *Astragalus membranaceus* (Fisch.) Bge. Var. mongholicus (Bge.) Hsiao | Root | ChP |
|  |  | Danggui | 当归 | *Angelica sinensis* (Oliv.) Diels | Root | ChP |
|  |  | Mohanlian | 墨旱莲 | *Eclipta aponicas* L. | Whole plant | ChP |
|  |  | Cangzhu* | 苍术 | *Atractylodes lancea* (Thunb.) DC. | Rhizome | ChP |
|  |  | Honghua | 红花 | *Carthamus tinctorius* L. | Flower | ChP |
|  |  | Chisao* | 赤芍 | *Paeonia lactiflora* Pall | Root | ChP |
|  |  | Taoren* | 桃仁 | *Prunus persica (L.)* Batsch | Seed | ChP |
|  |  | Niuxi | 牛膝 | *Achyranthes bidentata* Bl. | Root | ChP |
|  |  | Chuanxiong | 川芎 | *Ligusticum chuanxiong* Hort. | Rhizome | ChP |
|  |  | Zhiqiao | 枳壳 | *Citrus aurantium* L. | Immature fruit | ChP |
|  |  | Jiegeng | 桔梗 | *Platycodon grandiflorum* (Jacq.) A. DC | Root | ChP |
|  |  | Mahan | 麻罕 | Unknown | - | NO |
| 22 | Yi Shen Jian Gu Tablet | Qiannianjian | 千年健 | *Homalomena occulta* (Lour.) Schott | Rhizome | ChP |
|  |  | Honghua | 红花 | *Carthamus tinctorius* L. | Flower | ChP |
|  |  | Sharen* | 砂仁 | *Amomum villosum* Lour | Fruit | ChP |
|  |  | Danggui | 当归 | *Angelica sinensis* (Oliv.) Diels | Root | ChP |
|  |  | Danshen | 丹参 | *Scrophularia ningpoensis* Hemsl. | Rhizome and root | ChP |
|  |  | Bajitian | 巴戟天 | *Morinda officinalis* How | Root | ChP |
|  |  | Duzhong | 杜仲 | *Eucommia ulmoides* Oliv. | Bark | ChP |
|  |  | Yinyanghuo* | 淫羊藿 | *Epimedium brevicornum* Maxim. | Leaf | ChP |
|  |  | Baizhu | 白术 | *Atractylodes macrocephala* Koidz. | Rhizome | ChP |
|  |  | Sanqi | 三七 | *Panax notoginseng* (Burk.) F. H. Chen | Rhizome and root | ChP |
|  |  | Chuanxiong | 川芎 | *Ligusticum chuanxiong* Hort. | Rhizome | ChP |
|  |  | Renshen | 人参 | *Panax ginseng* C. A. Mey. | Rhizome and root | ChP |
|  |  | Shudihuang | 熟地黄 | *Rehmannia glutinosa* Libosch. | Root tuber | ChP |
|  |  | Heshouwu | 何首乌 | *Polygonum multiflorum* Thunb. | Root tuber | ChP |
|  |  | Nvzhenzi | 女贞子 | *Ligustrum lucidum* Ait. | Fruit | ChP |
|  |  | Gancao | 甘草* | *Glycyrrhiza uralensis* Fisch. | Rhizome and root | ChP |
| 23 | Yin Qing Capsule | Jinyinhua | 金银花 | *Lonicera japonica* Thunb. | Bud and opening flower | ChP |
|  |  | Huangqin | 黄芩 | *Scutellaria baicalensis* Georgi | Root | ChP |
|  |  | Yuxingcao | 鱼腥草 | *Houttuynia cordata* Thunb. | Whole plant | ChP |
|  |  | Sanqiye | 三七叶 | *Panax notoginseng* (Burk.) F. H. Chen | Stem and leaf | SGXP |
| 24 | Zhu Zi Gan Tai Capsule | Zhuzicao | 珠子草 | *Phyllanthus niruri* Linn | Whole plant | SYNP |
| 25 | Xiao Jie An Oral liquid | Yimucao | 益母草 | *Leonurus aponicas*Houtt | Whole plant | ChP |
|  |  | Jixueteng | 鸡血藤 | *Spatholobus suberectus* Dunn | Cane | ChP |
|  |  | Lianqiao | 连翘 | *Forsythia* suspensa | Fruit | ChP |
|  |  | Tufuling | 土茯苓 | *Smilax Glabra* Roxb | Rhizome | ChP |
|  |  | Gonglaomu* | 功劳木 | *Mahonia bealei* (Fort.) Carr. | Stem | ChP |
|  |  | Sanchaku | 三叉苦 | *Evodia lepta* (Spreng.) Merr. | Whole plant | SGXP |
| 26 | Polygonum Cuspidatum and Aluminite Liniment | Huzhang | 虎杖 | *Polygonum cuspidatum* Sieb.et Zucc | Rhizome and root | ChP |
|  |  | Baifan | 白矾 | KAI(SO_4_)_2_·12H_2_O | Mineral | ChP |
|  |  | Shigao | 石膏 | CaSO_4_∙2H_2_O | Mineral | ChP |
| 27 | Ya Jiao Ha Dun Powder | Tengkushen | 藤苦参 | *Streptocaulon juventas* (Lour.) Merr. | Root | SYNP |
|  |  | Jiangenshu | 箭根薯 | *Tacca chantrieri* Andre | Root tuber | SYNP |
|  |  | Yangerjugen | 羊耳菊根 | *Inula cappa* (Buch -Ham) DC | Root | SYNP |
|  |  | Kudonggua | 苦冬瓜 | *Benincasa hispida* (Thunb.) Cogn. | Fruit | SYNP |
|  |  | Xiaobaibu | 小百部 | Unknown |  | NO |
|  |  | Manjingzijingye | 蔓荆子茎及叶 | *Vitex trifolia Linn*.var.sirnplici folia Chain. | Stem and leaf | NO |
| 28 | Ru Bi Qing Capsule | Chaihu* | 柴胡 | *Bupleurum chinese* DC. | Root | ChP |
|  |  | Qingpi | 青皮 | *Citrus reticulata* Blanco | Immature fruit peel | ChP |
|  |  | Gualoupi* | 瓜萎皮 | T*richosanthes kirikrwii* Maxim. | Fruit peel | ChP |
|  |  | Pugongying* | 蒲公英 | *Taraxacum mongolicum* Hand. -Mazz. | Whole plant | ChP |
|  |  | Shancigu* | 山慈菇 | *Gremastrxi appendiculata* (Don) Makino | Pseudobulb | ChP |
|  |  | Lujiaoshuang* | 鹿角霜 | *Cervus elaphus* Linnaeus | Antler | ChP |
|  |  | Tumuxiang | 土木香 | *Inula helenium* L. | Root | ChP |
|  |  | Tubeimu | 土贝母 | *Bolbostemma paniculatum* (Maxim.) Franquet | Root tuber | ChP |
|  |  | Xiakucao | 夏枯草 | *Prunella vulgaris* L. | Ear of fruit | ChP |
|  |  | Dongchongxiacao | 冬虫夏草 | *Cordyceps sinensis* (BerK.) Sacc. | Fungus complex | ChP |
|  |  | Chonglou* | 重楼 | *Paris polyphylla* Smith var. chinenisi (Franch) Hara | Rhizome | ChP |
|  |  | Wuqichaoyangcao | 五气朝阳草 | *Geum aleppicum* Thumb.var.chinese Bolle | Whole plant | SYNP |
|  |  | Danggui | 当归 | *Angelica sinensis* (Oliv.) Diels. | Root | ChP |

Note: * means that medicine has more origins of species. DPM: Dai patent medicine; Ref. : Reference; ChP: Chinese Pharmacopoeia; SYNP: Standards for Chinese medicinal materials in Yunnan Province;SGZP: Standards for Chinese medicinal materials in Guizhou Province (2009); SHNP: Standards for Chinese medicinal materials in Hunan Province (2010); SGXP: Standards for Chinese medicinal materials in Guangxi Province (2013)

;
