# Supplementary material for: Policies and Problems of Modernizing Ethnomedicine in China: A Focus on the Yi and Dai Traditional Medicines of Yunnan Province
Source: Evid Based Complement Alternat Med. 2020 Aug 14;2020:1023297. doi: 10.1155/2020/1023297 (PMC7443223; doi:10.1155/2020/1023297)
Supplement: Supplementary Materials — Tables S1 and S2: in this article, all the ethnic patent medicines (EPMs) produced in Yunnan Province were collected. The information of these EPMs is obtained from the National Medical Products Administration of China, and all EPMs listed in the article have been checked according to open drug instructions. Table S1 and Table S2 provide the following information including the drug name, Chinese name, approval number, clinical indications, dosage form, and prescription status of Yi patent medicines (YPMs) and Dai patent medicines (DPMs). Some of these data supported the results of “clinical indications of Yi and Dai medicines” and Figure 1 in this article. Tables S3 and S4: in this article, the investigation focused on the composition of each EPM, the types of medicinal resources and medicinal parts, and quality standards for medicinal materials used in EPMs, including botanical, animal, and mineral resources. All these data are listed as two tables, showing separately the composition information and quality standards in DPMs (Table S3) and YPMs (Table S4). The information includes the drug name and pinyin name, Chinese name, scientific name, and medicinal parts of medicinal materials used in EPMs, and also contains the quality standard information of medicinal materials which can be regarded as the most important key supporting data for this article. [file 1023297.f1.zip › other materials/table S4.docx]

**Table S4 Composition information and Quality standards in YPMs**

| No. | Drug Name | Pinying name | Chinese name | Scientific name | Medicinal parts | Ref |
| --- | --- | --- | --- | --- | --- | --- |
| 1 | Bai Bei Yi Fei Capsule (BBYFC) | Baiji | 白及 | *Bletilla striata* (Thunb.) Reichb.f. | Tuber | ChP |
|  |  | Zhebeimu | 浙贝母 | *Fritillaria thunbergii* Miq | Bulb | ChP |
|  |  | Jiegeng | 桔梗 | *Platycodon grandiflorum* (Jacq.) A. DC | Root | ChP |
|  |  | Baibu* | 百部 | *Stemona sessilifolia* (Miq.) Miq. | Root tuber | ChP |
|  |  | Baihe* | 百合 | *Lilium lancifolium* Thunb. | Succulent scales | ChP |
|  |  | Ziyuan | 紫菀 | *Aster tataricus* L. f. | Rhizome and root | ChP |
|  |  | Sanqi | 三七 | *Panax notoginseng* (Burk.) F. H. Chen | Rhizome and root | ChP |
|  |  | Gonglaomu* | 功劳木 | *Mahonia bealei* (Fort.) Carr. | Stem | ChP |
|  |  | Gancao* | 甘草 | *Glycyrrhiza uralensis* Fisch. | Rhizome and root | ChP |
|  |  | Haifushi | 海浮石 | *Costazia aculeate* Canu et Bassler | skeleton | SHLP |
| 2 | Chang Shu Tablet  (CST) | Root of Gugongguo | 固公果根 | Unknown | - | NO |
| 3 | Chang Shu Zhi Xie Capsule (CSZXC) | Sharen* | 砂仁 | *Amomum villosum* Lour. | Fruit | ChP |
|  |  | Renshen | 人参 | *Panax ginseng* C. A. Mey. | Rhizome and root | ChP |
|  |  | Shanyao | 山药 | *Dioscorea aponica* Thunb. | Rhizome | ChP |
|  |  | Cangzhu* | 苍术 | *Atractylodes lancea* (Thunb.) DC. | Rhizome | ChP |
|  |  | Xiaohuixiang | 小茴香 | *Foeniculum vulgare* Mill. | Fruit | ChP |
|  |  | Roudoukou | 肉豆蔻 | *Myristica fragrans* Houtt. | Seed | ChP |
|  |  | Hezi | 诃子 | *Terminalia chebula* Retz. | Fruit | ChP |
|  |  | Gancao* | 甘草 | *Glycyrrhiza uralensis* Fisch. | Rhizome and root | ChP |
|  |  | Jiaoshanzha* | 焦山楂 | *Crataegus pinnatifida* Bunge.var.major N. E. Br | Fruit | ChP |
|  |  | Jishiteng | 鸡矢藤 | *Paederia scandens* (Lour.) Merr. | Whole plant | SHNP |
| 4 | Chang Wei Shu Capsule (CWSC) | Caoguo | 草果 | Amomum tsao-ko Crevost et Lemaire | Fruit | ChP |
|  |  | Muxiang | 木香 | Aucklandia lappa Decne. | Root | ChP |
|  |  | Zhizhuxiang | 蜘蛛香 | Valeriana jatamansi Jones. | Rhizome and root | ChP |
|  |  | Zidiyu | 紫地榆 | Geranium strictipes R. Knuth | Root | NO |
|  |  | Caoxuejie | 草血竭 | Polygonum paleaceum Wall.ex Hook. | Rhizome | SYNP |
| 5 | Chuan Luo Tong Capsule (CLTC) | Jinqiaomai | 金荞麦 | *Fagopyrum dibotrys* (D. Don) Hara | Rhizome | ChP |
|  |  | Renshen | 人参 | *Panax ginseng* C. A. Mey. | Rhizome and root | ChP |
|  |  | Gejie | 蛤蚧 | *Gekko gecko* Linnaeus | Body | ChP |
|  |  | Dilong* | 地龙 | *Pheretima aspergillum* (E. Perrier) | Insect body | ChP |
|  |  | Chanshu | 蟾酥 | *Bufo bufo gargarizans* Cantor | Secretion | ChP |
|  |  | Zhebeimu | 浙贝母 | *Fritillaria thunbergii* Miq | Bulb | ChP |
|  |  | Mahuang | 麻黄 | *Ephedra sinica* Stapf | Herbaceous stalk | ChP |
|  |  | Kuxinren* | 苦杏仁 | *Prunus armeniaca* L. var. ansu Maxim | Seed | ChP |
|  |  | Gancao* | 甘草 | *Glycyrrhiza uralensis* Fisch. | Rhizome and root | ChP |
|  |  | Jigen | 鸡根 | *Polygala arillata* Buch. Ham. ex D .Dom | Rhizome and root | SYNP |
|  |  | Ziheche | 紫河车 | - |  | NO |
| 6 | Shu Lie An Capsule (SLAC) | Dafabiao | 大发表 | *Campylotropis trigonoclada* (Franch.) A.K.Schindl. | Whole plant | SYNP |
| 7 | Dan Deng Tong Nao Capsule (DDTNC) | Danshen | 丹参 | *Salvia miltiorrhiza* Bunge. | Rhizome and root | ChP |
|  |  | Dengzhanxixin | 灯盏细辛 | *Erigeron breviscapus* (Vaniot) Hand. -Mazz. | Whole plant | ChP |
|  |  | Chuanxiong | 川芎 | *Ligusticum chuanxiong* Hort. | Rhizome | ChP |
|  |  | Gegen | 葛根 | *Pueraria lobata* (Willd.) Ohwi | Root | ChP |
| 8 | Dan E Fu Kang Ointment (DEFKO) | Ezhu | 莪术 | *Curcuma phaeocaulis* Val. | Rhizome | ChP |
|  |  | Sanqi | 三七 | *Panax notoginseng* (Burk.) F. H. Chen | Rhizome and root | ChP |
|  |  | Chisao* | 赤芍 | *Paeonia lactiflora* Pall | Root | ChP |
|  |  | Danggui | 当归 | *Angelica sinensis* (Oliv.) Diels | Root | ChP |
|  |  | Sanleng | 三棱 | *Sparganium stoloniferum* Buch. -Ham. | Tuber | ChP |
|  |  | Xiangfu | 香附 | *Cyperus rotundus* L. | Rhizome | ChP |
|  |  | Yanhusuo | 延胡索 | *Corydalis yanhusuo* W. T. Wang | Tuber | ChP |
|  |  | Gancao* | 甘草 | *Glycyrrhiza uralensis* Fisch. | Rhizome and root | ChP |
|  |  | Zidanshen | 紫丹参 | *Salvia yunnanensis* C.H. Wright | Root | SYNP |
|  |  | Zhuyecaihu | 竹叶柴胡 | *Bupleurum marginatum* Wallich ex de Candolle | Whole plant | SHNP |
| 9 | Danshen Yi Xin Capsule (DYXC) | Sanqi | 三七 | *Panax notoginseng* (Burk.) F. H. Chen | Rhizome and root | ChP |
|  |  | Dengzhanxixin | 灯盏细辛 | *Erigeron breviscapus* (Vaniot) Hand. -Mazz | Whole plant | ChP |
|  |  | Heshouwu | 何首乌 | *Polygonum multiflorum* Thunb. | Root tuber | ChP |
|  |  | Yanhusuo | 延胡索 | *Corydalis yanhusuo* W. T. Wang | Tuber | ChP |
|  |  | Zidanshen | 紫丹参 | *Salvia yunnanensis* C.H. Wright | Root | SYNP |
|  |  | Huixincao | 回心草 | *Rhodobryum* *giganteum* (Hook.) Par. | Whole plant | SYNP |
| 10 | Dan Wei Kang Capsule (DWKC) | Ziyedan | 青叶胆 | *Swertia mileensis* T. N. He et W. L. Shi | Whole plant | ChP |
|  |  | Zhiqiao | 枳壳 | *Citrus aurantium* L. | Immature fruit | ChP |
|  |  | Baishao | 白芍 | *Paeonia lactiflora* Pall. | Root | ChP |
|  |  | Zexie | 泽泻 | *Alisma orientalis* (Sam.) Juzep. | Tuber | ChP |
|  |  | Fuling | 茯苓 | *Poria cocos* (Schw.) Wolf | Sclerotia | ChP |
|  |  | Yinchen* | 茵陈 | *Artemisia scoparia* Waldst. et Kit. | Whole plant | ChP |
|  |  | Danzhuye | 淡竹叶 | *Lophatherum gracile* Brongn. | Stem and leaf | ChP |
|  |  | Dengxincao | 灯心草 | *Juncus effusus* L. | Pith of stem | ChP |
|  |  | Zhuyecaihu | 竹叶柴胡 | *Bupleurum marginatum* Wallich ex de Candolle | Whole plant | SHNP |
| 11 | Deng Yin Nao Tong Capsule (DYNTC) | Xinanhuangqin | 西南黄芩 | *Scutellaria amoena* C.H. Wright. | Root, stem and leaf | SGZP |
|  |  | Dengzhanxixin | 灯盏细辛 | *Erigeron breviscapus* (Vaniot) Hand. -Mazz. | Whole plant | ChP |
|  |  | Yinxinye | 银杏叶 | *Ginkgo biloba* L. | Leaf | ChP |
|  |  | Sanqi | 三七 | *Panax notoginseng* (Burk.) F. H. Chen | Rhizome and root | ChP |
|  |  | Manshanxiang* | 满山香 | *Schisandra propinqua* (Wall.) Bail.var.intermedia A.C. Smith | Whole plant | SYNP |
| 12 | E Qiu Qi Capsule (EQQC) | Laoguancao* | 老鹳草 | *Erodium stephanianum* Willd. | Whole plant | ChP |
|  |  | Cangzhu* | 苍术 | *Atractylodes lancea* (Thunb.) DC. | Rhizome | ChP |
|  |  | Caoxuejie | 草血竭 | *Polygonum paleaceum* Wall.ex Hook. | Rhizome | SYNP |
|  |  | Yantuo* | 岩陀 | *Rodgersia sambucifolia* Hemsl | Rhizome | SHNP |
| 13 | Fan Teng Zhi Injection (FTZI) | Huangteng | 黄藤 | *Fibraurea recisa* Pierre. | Cane | ChP |
|  |  | Baifan | 白矾 | KAI(SO_4_)_2_·12H_2_O | Mineral | ChP |
|  |  | Cishizhi | 赤石脂 | Al_4_(Si_4_O_10_) (OH)_8_∙4H_2_O | Mineral | ChP |
| 14 | Fufang Dahongpan Zhi Xue Capsule (FFDZXC) | Shidi | 柿蒂 | *Diospyros kaki* Thunb. | Persistent calyx | ChP |
|  |  | Dahongpao | 大红袍 | *Campylotropis hirtella* (Franchet) Schindler | Root | SHNP |
| 15 | Fufang Luxiancao Granular (FFLG) | Jiuxiangchong | 九香虫 | *Aspongopus chinensis* Dallas | 全体 | ChP |
|  |  | Kushen | 苦参 | *Sophora flavescens* Ait. | Root | ChP |
|  |  | Tianhuafen | 天花粉 | *Trichosanthes kirilowii* Maxim | Root | ChP |
|  |  | Tufuling | 土茯苓 | *Smilax glabra* Roxb. | Rhizome | ChP |
|  |  | Luxiancao* | 鹿仙草 | *Balanophora harlandii* Hook.f. | Whole plant | SYNP |
|  |  | Huangyaozi | 黄药子 | *Dioscorea bulbifera* L. | Tuber | SGDP |
| 16 | Fufang Qinghao Spray  (FFQS) | Qinghao | 青蒿 | *Artemisia annua* L. | Whole plant | ChP |
|  |  | Huangqin | 黄芩 | *Scutellaria baicalensis* Georgi | Root | ChP |
|  |  | Qingyedan | 青叶胆 | *Swertia mileensis* T. N. He et W. L. Shi | Whole plant | ChP |
|  |  | Sanqi | 三七 | *Panax notoginseng* (Burk.) F. H. Chen | Rhizome and root | ChP |
|  |  | Dahuangteng | 大黄藤 | *Fibraurea recisa* Pierre | Cane | NO |
| 17 | Fu Yi Shen Alcohol (FYSA) | Fuling | 茯苓 | *Poria cocos* (Schw.) Wolf | Sclerotia | ChP |
|  |  | Juanbai* | 卷柏 | *Selaginella pulvinata* (Hook.et Grev.) Maxim | Whole plant | ChP |
|  |  | Danggui | 当归 | *Angelica sinensis* (Oliv.) Diels | Root | ChP |
|  |  | Qiannianjian | 千年健 | *Homalomena occulta* (Lour.) Schott | Rhizome | ChP |
|  |  | Tianma | 天麻 | *Gastrodia elata* Bl. | Tuber | ChP |
|  |  | Dangshen* | 党参 | *Codonopsis pilosula* (Franch.) Nannf. | Root | ChP |
|  |  | Huangqi* | 黄芪 | *Astragalus membranaceus* (Fisch.) Bge. Var. mongholicus (Bge.) Hsiao | Root | ChP |
|  |  | Zhujieshen | 竹节参 | *Panax japonicus* C. A. Mey. | Rhizome | ChP |
|  |  | Sanqi | 三七 | *Panax notoginseng* (Burk.) F. H. Chen | Rhizome and root | ChP |
|  |  | Heimayi | 黑蚂蚁 | *Polyrhachis dives* Smith | Insect body | SYNP |
| 18 | Gan Dan Qing Capsule (GDQC) | Jinqiancao | 金钱草 | *Lysimachia christinae* Hance | Whole plant | ChP |
|  |  | Longdan | 龙胆* | *Gentiana manshurica* Kitage. | Rhizome and root | ChP |
|  |  | Dahuang | 大黄 | *Rheum palmatum* L. | Rhizome and root | ChP |
|  |  | Huanglian* | 黄连 | *Coptis chinensis* Franch. | Rhizome | ChP |
|  |  | Yanhusuo | 延胡索 | *Corydalis yanhusu*o W. T. Wang | Tuber | ChP |
|  |  | Jineijing | 鸡内金 | *Gallus gallus* domesticus Brisson | Chicken Sachet | ChP |
|  |  | Zheshi | 赭石 | Fe_2_O_3_ | Mineral | ChP |
|  |  | Wuzhuyu* | 吴茱萸 | *Evodia rutaecarpa* (Juss.) Benth. | Fruit | ChP |
|  |  | Zhuzongcao* | 猪鬃草 | *Adiantum capillus*-veneris L. | Whole plant | SGZP |
| 19 | Gu Feng Ning Capsule (GFNC) | Chonglou* | 重楼 | *Paris polyphylla* Smith var. chinenisi (Franch) Hara | Rhizome | ChP |
|  |  | Huangqi* | 黄芪 | *Astragalus membranaceus* (Fisch.) Bge. Var. mongholicus (Bge.) Hsiao | Root | ChP |
|  |  | Chuanniuxi | 川牛膝 | *Cyathula officinalis* Kuan | Root | ChP |
|  |  | Honghua | 红花 | *Carthamus tinctorius* L. | Flower | ChP |
|  |  | Gejie | 蛤蚧 | *Gekko gecko* Linnaeus | 全体 | ChP |
|  |  | Dilong | 地龙 | *Pheretima aspergillum* (E. Perrier) | Insect body | ChP |
|  |  | Shenjingcao | 伸筋草 | *Pheretima pectinifera* Michaelsen | Whole plant | ChP |
|  |  | Xuduan | 续断 | *Dipsacus asper* Wall.ex Henry | Root | ChP |
|  |  | Yexiahua | 叶下花 | *Ainsliaea pertyoides* Franch.var.albo-tomentosa Beauv. | Whole plant | SYNP |
|  |  | Huobahuagen | 火把花根 | *Tripterygium hypoglaucum* (Levl.) Hutch | Root | SYNP |
|  |  | Zidanshen | 紫丹参 | *Salvia yunnanensis* C.H. Wright | Root | SYNP |
|  |  | Yunweiling | 云威灵 | *Duhaldea nervosa* (Wallich ex Candolle) A. Anderberg | Rhizome and root | SHNP |
| 20 | He Wei Zhi Tong Capsule (HWZTC) | Jinqiaomai | 金荞麦 | *Fagopyrum dibotrys* (D. Don) Hara | Rhizome | ChP |
|  |  | Huanglian | 黄连 | *Coptis chinensis* Franch. | Rhizome | ChP |
|  |  | Sharen* | 砂仁 | *Amomum villosum* Lour. | Fruit | ChP |
|  |  | Yanhusuo | 延胡索 | *Corydalis yanhusuo* W. T. Wang | Tuber | ChP |
|  |  | Muxiang | 木香 | *Aucklandia lappa* Decne. | Root | ChP |
|  |  | Guanzhong | 管仲 | *Potentilla fulgens* Wall.ex Hook. | Root | SYNP |
|  |  | Dahongpao | 大红袍 | *Campylotropis hirtella* (Franchet) Schindler | Root | SHNP |
|  |  | Jishiteng | 鸡矢藤 | *Paederia scandens* (Lour.) Merr. | Whole plant | SHNP |
| 21 | Wen Zhong He Wei Capsule (WZHWC) | Tumuxiang | 土木香 | *Inula helenium* L. | Root | ChP |
|  |  | Gancao* | 甘草 | *Glycyrrhiza uralensis* Fisch. | Rhizome and root | ChP |
|  |  | Xiaoerfutongcao | 小儿腹痛草 | *Swertia patens* Burk. | Whole plant | SYNP |
|  |  | Yangerju | 羊耳菊 | *Inula cappa* (Buch -Ham) DC. | Whole plant | SYNP |
| 22 | Huzhang Shang Tong Tincture (HSTT) | Gouteng* | 钩藤 | Uncaria rhynchphylla (Miq.) Miq.ex | Stem and branch with hook | ChP |
|  |  | Huzhang | 虎杖 | *Polygonum cuspidatum* Sieb.et Zucc | Rhizome and root | ChP |
|  |  | Qianzhiyan | 千只眼 | *Murraya tetramera* Huang | Leaf | SYNP |
|  |  | Dafahan | 大发汗 | *Millettia bonatiana* Pamp. | Root | SYNP |
|  |  | Heiniuxi | 黑牛膝 | *Piper boehmeriaefolium* (Miq.) C.DC.var.tonkinense C.DC. | Rhizome and root | SYNP |
|  |  | Damayao | 大麻药 | *Raw Radix* Dolichosae | - | NO |
| 23 | Hu Zhang leaf Capsule (HZYC) | Leaf of Huzhang | 虎杖叶 | *Polygonum cuspidatum* Sieb.et Zucc. | Leaf | SYNP |
| 24 | Huoxiang Wan Ying Powder (HWYP) | Houpu* | 厚朴 | *Magnolia officinalis* Rehd. Et Wils. | Bark, root bark and branch bark | ChP |
|  |  | Chenpi | 陈皮 | *Citrus aponicas* Blanco | Pericarp | ChP |
|  |  | Cangzhu* | 苍术 | *Atractylodes lancea* (Thunb.) DC. | Rhizome | ChP |
|  |  | Guanghuoxiang | 广藿香 | *Pogostemon cablin* (Blanco) Benth. | Whole plant | ChP |
|  |  | Dazao | 大枣 | *Ziziphus aponi* Mill. | Fruit | ChP |
|  |  | Sharen* | 砂仁 | *Amomum villosum* Lour. | Fruit | ChP |
|  |  | Wuzhuyu | 吴茱萸 | *Evodia rutaecarpa* (Juss.) Benth. | Fruit | ChP |
|  |  | Dingxiang | 丁香 | *Eugenia caryophllata* Thunb. | Bud | ChP |
|  |  | Ganjiang | 干姜 | *Zingiber officinale* Rosc. | Rhizome | ChP |
|  |  | Baihujiao | 白胡椒 | *Piper nigrun* L． | Fruit | SSHP |
|  |  | Zouyexiangru | 皱叶香薷 | Unknown | - | NO |
| 25 | Jiang Zhi Tong Mai Capsule (JZTMC) | Juemingzi | 决明子 | *Cassia obtusifolia* L. | Seed | ChP |
|  |  | Jianghuang | 姜黄 | *Curcuma longa* L. | Rhizome | ChP |
|  |  | Zexie | 泽泻 | *Alisma orientalis* (Sam.) Juzep. | Tuber | ChP |
|  |  | Sanqi | 三七 | *Panax notoginseng* (Burk.) F. H. Chen | Rhizome and root | ChP |
|  |  | Tiexiancao | 铁线草 | *Cynodon dactylon* (L.) Pets. | Whole plant | SYNP |
| 26 | Kang Shen Granular (KSG) | Lianqiancao | 连钱草 | *Glechoma longituba* (Nakai)Kupr. | Whole plant | ChP |
|  |  | Rendongteng | 忍冬藤 | *Lonicera japonica* Thunb. | Stem and branch | ChP |
|  |  | Shiwei | 石韦 | *Pyrrosia sheareri* (Bak.) Ching | Rhizome and root | ChP |
|  |  | Baimaogen | 白茅根 | *Imperata aponicas* Beauv. Var. major (Nees) C. E. Hubb. | Rhizome | ChP |
|  |  | Shichangpu | 石菖蒲 | *Acorus tatarinowii* Schott | Rhizome | ChP |
|  |  | Gegen | 葛根 | *Pueraria lobata* (Willd.) Ohwi | Root | ChP |
|  |  | Qiancao | 茜草 | *Rubia cordifolia* L. | Rhizome and root | ChP |
|  |  | Aiye | 艾叶 | *Artemisia argyi* Levl. Et Vant. | Leaf | ChP |
|  |  | Shengjiang | 生姜 | *Zingiber officinale* Rosc. | Rhizome | ChP |
|  |  | Chenpi | 陈皮 | *Citrus aponicas* Blanco | Pericarp | ChP |
|  |  | Laoguancao | 老鹳草 | *Erodium stephanianum* Willd. | Whole plant | ChP |
|  |  | Shuiwugong | 水蜈蚣 | *Kyllinga brevifolia* Rottb | Whole plant | SGZP |
| 27 | Ke Tan Oral liquid (KTL) | Cheqiancao* | 车前草 | *Plantago asiatica* L. | Whole plant | ChP |
|  |  | Qianliguang | 千里光 | *Senecio scandens* Buch. -Ham. | Whole plant | ChP |
|  |  | Yinsuqiao | 罂粟壳 | *Papaver somniferum* L. | Nutshell | ChP |
|  |  | Jiegeng | 桔梗 | *Platycodon grandiflorum* (Jacq.) A. DC | Root | ChP |
|  |  | Pugongying* | 蒲公英 | *Taraxacum mongolicum* Hand. | Whole plant | ChP |
| 28 | Li Dan Jie Du Capsule  (LDJDC) | Longdan* | 龙胆 | *Gentiana manshurica* Kitage. | Rhizome and root | ChP |
|  |  | Tumuxiang | 土木香 | *Inula helenium* L. | Root | ChP |
|  |  | Baishao | 白芍 | *Paeonia lactiflora* Pall. | Root | ChP |
|  |  | Xiaoerfutongcao | 小儿腹痛草 | *Swertia patens* Burk. | Whole plant | SYNP |
|  |  | Jishiteng | 鸡矢藤 | *Paederia scandens* (Lour.) Merr. | Whole plant | SHNP |
| 29 | Lingdancao Oral liquid (LL) | Coulingdancao | 臭灵丹草 | *Laggera pterodonta* (DC.) Benth. | Whole plant | ChP |
| 30 | Long Jing Tong Lin Capsule (LJTLC) | Longdan | 龙胆 | *Gentiana manshurica* Kitage. | Rhizome and root | ChP |
|  |  | Yuxingcao | 鱼腥草 | *Houttuynia cordata* Thunb. | Whole plant | ChP |
|  |  | Jinqiancao | 金钱草 | *Lysimachia christinae* Hance | Whole plant | ChP |
|  |  | Huangqi* | 黄芪 | *Astragalus membranaceus* (Fisch.) Bge. Var. mongholicus (Bge.) Hsiao | Root | ChP |
|  |  | Niuhuang | 人工牛黄 | - | - | ChP |
|  |  | Dihuang | 地黄 | *Rehmannia glutinosa* Libosch. | Tuber | ChP |
|  |  | Zhizi | 栀子 | *Gardenia jasminoides* Ellis | Fruit | ChP |
|  |  | Fuling | 茯苓 | *Poria cocos* (Schw.) Wolf | Sclerotia | ChP |
|  |  | Zidanshen | 紫丹参 | *Salvia yunnanensis* C.H. Wright | Root | SYNP |
|  |  | Xiongdanfen | 熊胆粉 | *Selenaretos thibetanus* Cuvier | Bile | SYNP |
|  |  | Baihuasheshecao | 白花蛇舌草 | *Hedyotis diffusa* Willd. | Whole plant | SSXP |
|  |  | Zhuyecaihu | 竹叶柴胡 | *Bupleurum marginatum* Wallich ex de Candolle | Whole plant | SHNP |
| 31 | Lushuicao Capsule (LC) | Lushuicao | 露水草 | Unknowen | - | NO |
| 32 | Lvji Ke Chuan Granular (LKCG) | Tongguanteng | 通关藤 | *Marsdenia tenacissima* (Roxb.) Weight et Arn. | Cane | ChP |
|  |  | Gonglaomu* | 功劳木 | *Mahonia bealei* (Fort.) Carr. | Stem | ChP |
|  |  | Baiji | 白及 | *Bletilla striata* (Thunb.) Reichb.f. | Tuber | ChP |
|  |  | Huangjing* | 黄精 | *Polygonatum kingianum* Coll.et Hemsil. | Rhizome | ChP |
|  |  | Huzhang | 虎杖 | *Polygonum cuspidatum* Sieb.et Zucc | Rhizome and root | ChP |
|  |  | Tougucao | 透骨草 | *Speranskia tuberculata* (Bunge) Baillon | Whole plant | SYNP |
|  |  | Xiaolvji | 小绿芨 | unknown | - | NO |
|  |  | Jishiteng | 鸡矢藤 | *Paederia scandens* (Lour.) Merr. | Whole plant | SHNP |
| 33 | Mitonghua Granular (MG) | Mitonghua | 蜜桶花 | *Brandisia hancei* Hook.f. | Whole plant | SYNP |
| 34 | Niao Lu Kang Granular  (NLKG) | Yimucao | 益母草 | *Leonurus aponicas* Houtt. | Whole plant | ChP |
|  |  | Mohanliang | 墨旱莲 | *Eclipta aponicas* L. | Whole plant | ChP |
|  |  | Cheqiancao | 车前草 | *Plantago asiatica* L. | Whole plant | ChP |
|  |  | Shanyao | 山药 | *Dioscorea aponica* Thunb. | Rhizome | ChP |
|  |  | Jinqiancao | 金钱草 | *Lysimachia christinae* Hance | Whole plant | ChP |
|  |  | Gancao* | 甘草 | Glycyrrhiza uralensis Fisch. | Rhizome and root | ChP |
|  |  | Huangjing | 黄精 | *Polygonatum kingianum* Coll.et Hemsil. | Rhizome | ChP |
|  |  | Dengxincao | 灯心草 | *Juncus aponic* L. | Pith of stem | ChP |
| 35 | Niao Qing Shu Granular (NQSG) | Chonglou* | 重楼 | *Paris polyphylla* Smith var. chinenisi (Franch) Hara | Rhizome | ChP |
|  |  | Yejuhua | 野菊花 | *Chrysanthemum indicum* L. | Capitulum | ChP |
|  |  | Huzhang | 虎杖 | *Polygonum cuspidatum* Sieb.et Zucc | Rhizome and root | ChP |
|  |  | Cheqiancao | 车前草 | *Plantago asiatica* L. | Whole plant | ChP |
|  |  | Didancao | 地胆草 | *Elephantopus scaber* Linnaeus | Whole plant | SGDP |
|  |  | Shanmutong | 山木通 | *Clematis apiifolia* var. argentilucida (H. Leveille ＆ vaniot) W. T. Wang | Cane | SHNP |
| 36 | Ping Xuan Capsule (PXC) | Huangjing | 黄精 | *Polygonatum kingianum* Coll.et Hemsil. | Rhizome | ChP |
|  |  | Xianhecao | 仙鹤草 | *Agrimonia pilosa* Ledeb. | Whole plant | ChP |
|  |  | Sanqi | 三七 | *Panax notoginseng* (Burk.) F. H. Chen | Rhizome and root | ChP |
|  |  | Tianma | 天麻 | *Gastrodia elata* Bl. | Tuber | ChP |
|  |  | Chongmu | 楤木 | *Aralia chinensis* Linnaeus. | Root and root skin | SHNP |
|  |  | Zhuyangyang* | 猪殃殃 | *Galium aparine* L. var. echinospermun (Wallr.) Cuf. | Whole plant | SSCP |
|  |  | Wanzhangsheng | 万丈深 | *Crepis lignea* (Vaniot) Babcock | - | NO |
| 37 | Qiancao Nao Tong Oral liquid (QNTL) | Huweicao | 虎尾草 | *Lysimachia barystachys* Bunge | Whole plant | SYNP |
|  |  | Qianjinzhui | 千斤坠 | *Boschniakia himalaica* Hook.f.et Thoms. | Tuber | SYNP |
| 38 | Qing Chang Tong Bian Capsule (QCTBC) | Gouteng* | 钩藤 | *Uncaria rhynchphylla* (Miq.) Miq.ex | Stem and branch with hook | ChP |
|  |  | Caoguo | 草果 | *Amomum* tsao-ko Crevost et Lemaire. | Fruit | ChP |
|  |  | Matixiang | 马蹄香 | *Valeriana jatamansi* Jones | Rhizome and root | SYNP |
|  |  | Xiwanye | 洗碗叶 | *Solanum erianthum* D. Don | Stem | SYNP |
|  |  | Diwugong | 地蜈蚣 | *Arthromeris mairei* (Brause)Ching | Rhizome | SYNP |
| 39 | Rong Shuan Nao Tong Capsule (RSNTC) | Sanqi | 三七 | Panax notoginseng (Burk.) F. H. Chen | Rhizome and root | ChP |
|  |  | Gancao* | 甘草 | *Glycyrrhiza uralensis* Fisch. | Rhizome and root | ChP |
|  |  | Gejie | 蛤蚧 | *Gekko gecko* Linnaeus. | Body | ChP |
|  |  | Dilong | 地龙 | *Pheretima aspergillum* (E. Perrier) | Insect body | ChP |
|  |  | Dongchongxiacao | 冬虫夏草 | *Cordyceps sinensis* (BerK.) Sacc. | Fungus complex | ChP |
|  |  | Shanyao | 山药 | *Dioscorea aponica* Thunb. | Rhizome | ChP |
|  |  | Xuedan | 雪胆提取物 | *Hemsleya amabilis* Diels | Extract | NO |
| 40 | She Chang Zhi Xie Powder (SCZXP) | Pengruntu | 膨润土 | Montmorillonite | Mineral | SYNP |
|  |  | Yantuo* | 岩陀 | *Rodgersia sambucifolia* Hemsl | Rhizome | SHNP |
| 41 | Sha Mei Xiao Ke Capsule (SMXKC) | Niubangzi | 牛蒡子 | *Arctium lappa* L. | Fruit | ChP |
|  |  | Jiangchan | 僵蚕 | *Bombyx mori* Linnaeus. | Insect body | ChP |
|  |  | Baishao | 白芍 | *Paeonia lactiflora* Pall. | Root | ChP |
|  |  | Wumei | 乌梅 | *Prunus mume* (Sieh.) Sieb .et Zucc. | Fruit | ChP |
|  |  | Zhimu | 知母 | *Anemarrhena asphodeloides* Bunge. | Rhizome | ChP |
|  |  | Shencha | 肾茶 | *Clerodendranthus spicatus* (Thunberg) C. Y. Wu ex H. W. Li | Whole plant | SGZP |
|  |  | Shashen | 沙参 | *Adenophora tetraphylla* (Thunb.) Fisch | Root | NO |
| 42 | Shang Yi Aerosol (SYA) | Sanqi | 三七 | *Panax notoginseng* (Burk.) F. H. Chen | Rhizome and root | ChP |
|  |  | Chonglou* | 重楼 | *Paris polyphylla* Smith var. chinenisi (Franch) Hara | Rhizome | ChP |
|  |  | Gancao* | 甘草 | *Glycyrrhiza uralensis* Fisch. | Rhizome and root | ChP |
|  |  | Zhizi | 栀子 | *Gardenia jasminoides* Ellis | Fruit | ChP |
|  |  | Baiji | 白及 | *Bletilla striatac* (Thunb.) Reichb.f. | Tuber | ChP |
|  |  | Bohe | 薄荷脑 | DL-Menthol | - | ChP |
|  |  | Bingpian (Borneol) | 冰片 | C_10_H_18_O | - | ChP |
|  |  | Yuputaogen | 玉葡萄根 | *Amoelopsis delavayana* (Franch.) Planch. | Root | SYNP |
|  |  | Feilian | 蜚蠊 | *Periplaneta aponicas* Linnaeus | Insect body | SYNP |
|  |  | Qiyelian | 七叶莲 | *Schefflera venulosa* (Wight et Arn.) Harms | Whole plant | SYNP |
| 43 | Shen An Capsule (SAC) | Huangbai | 黄柏 | *Phellodendron chinese* Schneid. | Bark | ChP |
|  |  | Baimaogen | 白茅根 | *Imperata aponicas* Beauv. Var. major (Nees) C. E. Hubb. | Rhizome | ChP |
|  |  | Fuling | 茯苓 | *Poria cocos* (Schw.) Wolf | Sclerotia | ChP |
|  |  | Baizhu | 白术 | *Atractylodes macrocephala* Koidz. | Rhizome | ChP |
|  |  | Jinyinhua | 金银花 | *Lonicera japonica* Thunb. | Bud and opening flower | ChP |
|  |  | Zexie | 泽泻 | *Alisma orientalis* (Sam.) Juzep. | Tuber | ChP |
|  |  | Gancao* | 甘草 | *Glycyrrhiza uralensis* Fisch. | Rhizome and root | ChP |
|  |  | Dengxincao | 灯心草 | *Juncus aponic* L. | Pith of stem | ChP |
|  |  | Danzhuye | 淡竹叶 | *Lophatherum gracile* Brongn. | Stem and leaf | ChP |
|  |  | Huangqi* | 黄芪 | *Astragalus membranaceus* (Fisch.) Bge. Var. mongholicus (Bge.) Hsiao | Root | ChP |
|  |  | Shijiaocao | 石椒草 | *Boenninghausenia sessilicarpa* Levl. | Whole plant | SYNP |
|  |  | Shencha | 肾茶 | *Clerodendranthus spicatus* (Thunberg) C. Y. Wu ex H. W. Li | Whole plant | SGZP |
| 44 | Shen Qi Xin Shu Capsule (SQXSC) | Sanqi | 三七 | *Panax notoginseng* (Burk.) F. H. Chen | Rhizome and root | ChP |
|  |  | Danshen | 丹参 | *Salvia miltiorrhiza* Bunge. | Rhizome and root | ChP |
|  |  | Lingzhi | 灵芝 | *Ganoderma lucidum* (Leyss.ex Fr.) Karst． | Fruiting body | ChP |
|  |  | Gegen | 葛根 | *Pueraria lobata* (Willd.) Ohwi | Root | ChP |
|  |  | Honghua | 红花 | *Carthamus tinctorius* L. | Flower | ChP |
|  |  | Chuanxiong | 川芎 | *Ligusticum chuanxiong* Hort. | Rhizome | ChP |
|  |  | Jiangxiang | 降香 | *Dalbergia odorifera* T. Chen | Core material | ChP |
|  |  | Duzhong | 杜仲 | *Eucommia ulmoides* Oliv. | Bark | ChP |
|  |  | Baiwei* | 白薇 | *Cynanchum atratum* Bge | Rhizome and root | ChP |
|  |  | Gancao* | 甘草 | Glycyrrhiza uralensis Fisch. | Rhizome and root | ChP |
|  |  | Xianrenzhang | 仙人掌 | *Opuntia stricta* (Haw.) Haw. var. dilleniid (KerGawl.) Benson. | Stem | SYNP |
| 45 | Shijiaocao Ke Chuan Granular (SKCG) | Chenpi | 陈皮 | *Citrus aponicas* Blanco | Pericarp | ChP |
|  |  | Shichangpu | 石菖蒲 | *Acorus tatarinowii* Schott | Rhizome | ChP |
|  |  | Huzhang | 虎杖 | *Polygonum cuspidatum* Sieb.et Zucc | Rhizome and root | ChP |
|  |  | Tiandong | 天冬 | *Asparagus cochinchinensis* (Lour.) Merr. | Root tuber | ChP |
|  |  | Baibu* | 百部 | *Stemona sessilifolia* (Miq.) Miq. | Root tuber | ChP |
|  |  | Sangbaipi | 桑白皮 | *Morus alba* L. | Velamen | ChP |
|  |  | Coulingdancao | 臭灵丹草 | *Laggera pterodonta* (DC.) Benth. | Whole plant | ChP |
|  |  | Kuxinren* | 苦杏仁 | *Prunus armeniaca* L. var. ansu Maxim | Seed | ChP |
|  |  | Yuxingcao | 鱼腥草 | *Houttuynia cordata* Thunb. | Whole plant | ChP |
|  |  | Tongguanteng | 通关藤 | *Marsdenia tenacissima* (Roxb.) Weight et Arn. | Cane | ChP |
|  |  | Shijiaocao | 石椒草 | *Boenninghausenia* sessilicarpa Levl. | Whole plant | SYNP |
| 46 | Shu Mi Tong Capsule (SMTC) | Chuanmutong* | 川木通 | *Clematis armandi* Franch. | Cane | ChP |
|  |  | Gouteng* | 钩藤 | *Uncaria rhynchphylla* (Miq.) Miq.ex | Stem and branch with hook | ChP |
|  |  | Yejuhua | 野菊花 | *Chrysanthemum* *indicum* L. | Capitulum | ChP |
|  |  | Jinqiancao | 金钱草 | *Lysimachia christinae* Hance | Whole plant | ChP |
| 47 | Shu Wei Yao Alcohol (SWYA) | Ziqiguanzhong | 紫萁贯众 | *Osmunda aponica* Thunb. | \|  \| \| --- \|   Rhizome and petiole | ChP |
|  |  | Caoguo | 草果 | *Amomum tsao-ko* Crevost et Lemaire | Fruit | ChP |
|  |  | Rougui | 肉桂 | *Cinnamomum cassia* Presl | Bark | ChP |
|  |  | Bajiaohuixiang | 八角茴香 | *Illicium verum* Hook. F. | Fruit | ChP |
|  |  | Gancao* | 甘草 | *Glycyrrhiza uralensis* Fisch. | Rhizome and root | ChP |
| 48 | Tianhusui Yu Gan Tablet (TYGT) | Zhajiangcao | 酢浆草 | *Oxalis corniculata* Linn. | Whole plant | SYNP |
|  |  | Huzhangcao | 虎掌草 | *Anemone rivularis* Bunch. Ham. ex DC. | Root | SYNP |
|  |  | Tianhusui | 天胡荽 | *Hydrocotyle Sibthorpioides* Lam. | Whole plant | SYNP |
|  |  | Xinyefangfeng | 杏叶防风 | *Pimpinella candolleana* Wight et Arn. | Root or whole plant | SYNP |
| 49 | Tian Xiang Tincture (TXT) | Caowu | 草乌 | *Aconitum kusnezoffii* Reichb. | Root tuber | ChP |
|  |  | Tiannanxing* | 天南星 | *Arisaema erubescens* (Wall.) Schott. | Tuber | ChP |
|  |  | Honghua | 红花 | *Carthamus tinctorius* L. | Flower | ChP |
|  |  | Zicao* | 紫草 | *Arnebia euchroma* (Royle) Johnst. | Root | ChP |
|  |  | Anxixiang | 安息香 | *Styrax tonkinensis* (Pierre) Craib ex Hart. | Resin | ChP |
|  |  | Sanfensan* | 三分三 | *Anisodus acutangulus* C. Y. Wu et C. Chen, | Root | SYNP |
|  |  | Yunxiangcao | 芸香草 | *Cymbopogon distans* (Nees) Wats. | - | NO |
| 50 | Tian Jing Yang Yan Capsule (TJYYC) | Huangqi* | 黄芪 | *Astragalus membranaceus* (Fisch.) Bge. Var. mongholicus (Bge.) Hsiao | Root | ChP |
|  |  | Nvzhenzi | 女贞子 | *Ligustrum lucidum* Ait. | Fruit | ChP |
|  |  | Sanqi | 三七 | *Panax notoginseng* (Burk.) F. H. Chen | Rhizome and root | ChP |
|  |  | Xiaohongshen | 小红参 | *Rubia yunnanensis* Diels | Rhizome and root | SYNP |
|  |  | Dibanteng | 地板藤 | *Ficus tikoua* Bur. | Cane | SYNP |
|  |  | Yudaicao | 玉带草 | Unknown | - | NO |
| 51 | Tong Shu Capsule (TSC) | Dengzhanxixin | 灯盏细辛 | *Erigeron breviscapus* (Vaniot) Hand. -Mazz. | Whole plant | ChP |
|  |  | Zhizi | 栀子 | *Gardenia jasminoides* Ellis | Fruit | ChP |
|  |  | Zhuzishen | 珠子参 | *Panax aponicas* C. A. Mey. Var. major (Burk.) C. Y. Wu et K. M. Feng | Rhizome | ChP |
|  |  | Gancao* | 甘草 | *Glycyrrhiza uralensis* Fisch. | Rhizome and root | ChP |
|  |  | Chonglou* | 重楼 | *Paris polyphylla* Smith var. chinenisi (Franch) Hara | Rhizome | ChP |
|  |  | Sanqi | 三七 | *Panax notoginseng* (Burk.) F. H. Chen | Rhizome and root | ChP |
|  |  | Yuputaogen | 玉葡萄根 | *Amoelopsis delavayana* (Franch.) Planch. | Root | SYNP |
|  |  | Qiyelian | 七叶莲 | *Schefflera venulosa* (Wight et Arn.) Harms | Whole plant | SYNP |
| 52 | Tong Shu Kou Shuang Capsule (TSKSC) | Dahuang | 大黄 | *Rheum palmatum* L. | Rhizome and root | ChP |
|  |  | Zhishi | 枳实 | *Citrus aurantium* L. | Young fruit | ChP |
|  |  | Yinchen | 茵陈 | *Artemisia scoparia* Waldst. Et Kit. | Whole plant | ChP |
|  |  | Muzei | 木贼 | *Equisetum hiemale* L. | Whole plant | ChP |
|  |  | Qinjiao* | 秦艽 | *Gentiana macrophylla* Pall. | Root | ChP |
|  |  | Mudanpi | 牡丹皮 | *Paeonia suffruticosa* Andr. | Velamen | ChP |
|  |  | Danggui | 当归 | *Angelica sinensis* (Oliv.) Diels | Root | ChP |
|  |  | Xiakucao | 夏枯草 | *Prunella vulgaris* L. | Ear of fruit | ChP |
| 53 | Wei Fu Shu Capsule (WFSC) | Zhizhuxiang | 蜘蛛香 | *Valeriana jatamansi* Jones. | Rhizome and root | ChP |
|  |  | Banxia | 半夏 | *Pinellia aponic* (Thunb.) Breit | Root tuber | ChP |
|  |  | Huangqin | 黄芩 | *Scutellaria baicalensis* Georgi | Root | ChP |
|  |  | Bichenqie | 荜澄茄 | *Litsea cubeba* (Lour.) Pers. | Fruit | ChP |
|  |  | Pugongying* | 蒲公英 | *Taraxacum mongolicum* Hand. | Whole plant | ChP |
|  |  | Binlang | 槟榔 | *Areca catechu* L. | Seed | ChP |
|  |  | Ganjiang | 干姜 | *Zingiber officinale* Rosc. | Rhizome | ChP |
|  |  | Huanglian | 黄连 | *Coptis chinensis* Franch | Rhizome | ChP |
|  |  | Gancao* | 甘草 | *Glycyrrhiza uralensis* Fisch. | Rhizome and root | ChP |
|  |  | Zhiqiao | 枳壳 | *Citrus aurantium* L. | Immature fruit | ChP |
| 54 | Wen Ya Capsule (WYC) | Gejie | 蛤蚧 | *Gekko gecko* Linnaeus. | Body | ChP |
|  |  | Dilong | 地龙 | *Pheretima aspergillum* (E. Perrier). | Insect body | ChP |
|  |  | Dongchongxiacao | 冬虫夏草 | *Cordyceps sinensis* (BerK.) Sacc. | Fungus complex | ChP |
|  |  | Shijueming* | 石决明 | *Haliotis diversicolor* Reeve. | Shell | ChP |
|  |  | Juemingzi | 决明子 | *Cassia obtusifolia* L. | Seed | ChP |
|  |  | Gaotong | 膏桐 | *Jatropha curcas* L. | Root bark and stem bark | SYNP |
| 55 | Wujin Huo Xue Zhi Tong Capsule (WHXZTC) | Chisao* | 赤芍 | *Paeonia lactiflora* Pall | Root | ChP |
|  |  | Jinqiaomai | 金荞麦 | *Fagopyrum dibotrys* (D. Don) Hara | Rhizome | ChP |
|  |  | Daotihu* | 倒提壶 | *Delphinium yunnanense* Franch. | Whole plant and root | NO |
| 56 | Xiang Teng Capsule (XTC) | Tianxianteng | 天仙藤 | *Aristolochia debilis* Sieb. Et Zucc. | Whole plant | ChP |
|  |  | Huangqi* | 黄芪 | *Astragalus membranaceus* (Fisch.) Bge. Var. mongholicus (Bge.) Hsiao | Root | ChP |
|  |  | Huzhang | 虎杖 | *Polygonum cuspidatum* Sieb.et Zucc | Rhizome and root | ChP |
|  |  | Wuqichaoyangcao | 五气朝阳草 | *Geum aleppicum* Thumb.var.chinese Bolle | Whole plant | SYNP |
|  |  | Tougucao | 透骨草 | *Speranskia tuberculata* (Bunge) Baillon | Whole plant | SYNP |
|  |  | Xiaoerfutongcao | 小儿腹痛草 | *Swertia patens* Burk. | Whole plant | SYNP |
|  |  | Dazhuifeng | 大追风 | *Leycesteria aponic* Wall.var.stenosepala Rehd. | Whole plant | SYNP |
|  |  | Yunweiling | 云威灵 | *Duhaldea nervosa* (Wallich ex Candolle) A. Anderberg | Rhizome and root | SHNP |
|  |  | Wanzhangshen | 万丈深 | *Crepis lignea* (Vaniot) Babcock | - | NO |
| 57 | Yanhu Wei An Capsule (YWAC) | Baiji | 白及 | *Bletilla striata* (Thunb.) Reichb.f. | Tuber | ChP |
|  |  | Muxiang | 木香 | *Aucklandia lappa* Decne. | Root | ChP |
|  |  | Shengjiang | 生姜 | *Zingiber officinale* Rosc. | Rhizome | ChP |
|  |  | Haipiaoqiao | 海螵蛸 | *Sepiella maindronide* Rochebrune | Conch | ChP |
|  |  | Sharen* | 砂仁 | *Amomum villosum* Lour. | Fruit | ChP |
|  |  | Dazao | 大枣 | *Ziziphus aponi* Mill. | Fruit | ChP |
|  |  | Gancao* | 甘草 | *Glycyrrhiza uralensis* Fisch. | Rhizome and root | ChP |
|  |  | Yanhusuo | 延胡索 | *Corydalis yanhusuo* W. T. Wang | Tuber | ChP |
|  |  | Jishiteng | 鸡矢藤 | *Paederia scandens* (Lour.) Merr. | Whole plant | SHNP |
| 58 | Yan Lu Ru Kang Capsule (YLRKC) | Luxiancao* | 鹿衔草 | *Pyrola calliantha* H. Andres | Whole plant | ChP |
|  |  | Lujiaoshuang* | 鹿角霜 | *Cervus elaphus* Linnaeus | Antler | ChP |
|  |  | Yantuo* | 岩陀 | *Rodgersia sambucifolia* Hemsl | Rhizome | SHNP |
| 59 | Yan Shu Oral liquid  (YSL) | Xuanshen | 玄参 | *Scrophularia ningpoensis* Hemsl. | Root | ChP |
|  |  | Jiegeng | 桔梗 | *Platycodon grandiflorum* (Jacq.) A. DC | Root | ChP |
|  |  | Niubangzi | 牛蒡子 | *Arctium lappa* L. | Fruit | ChP |
|  |  | Shegan | 射干 | *Belamcanda chinensis* (L.) DC. | Rhizome | ChP |
|  |  | Chenpi | 陈皮 | *Citrus aponicas* Blanco | Pericarp | ChP |
|  |  | Gancao* | 甘草 | *Glycyrrhiza uralensis* Fisch. | Rhizome and root | ChP |
|  |  | Huzhangcao | 虎掌草 | *Anemone rivularis* Bunch. Ham. ex DC. | Root | SYNP |
|  |  | Wuxiangcao | 午香草 | *Anaphalis bulleyana* (J.F. Jeffr.) Chang | Whole plant | SYNP |
| 60 | Yi Xin Kang Capsule (YXKC) | Jixueteng | 鸡血藤 | *Spatholobus suberectus* Dunn | Cane | ChP |
|  |  | Dengzhanxixin | 灯盏细辛 | *Erigeron breviscapus* (Vaniot) Hand. -Mazz. | Whole plant | ChP |
|  |  | Jianghuang | 姜黄 | *Curcuma longa* L | Rhizome | ChP |
|  |  | Tumuxiang | 土木香 | *Inula helenium* L. | Root | ChP |
|  |  | Huzhang | 虎杖 | *Polygonum cuspidatum* Sieb.et Zucc | Rhizome and root | ChP |
|  |  | Wuqichaoyangcao | 五气朝阳草 | *Geum aleppicum* Thumb.var.chinese Bolle | Whole plant | SYNP |
|  |  | Tougucao | 透骨草 | *Speranskia tuberculata* (Bunge) Baillon | Whole plant | SYNP |
| 61 | Yu Mai Kou Yan Oral liquid (YMKYL) | Yuganzi | 余甘子 | *Phyllanthus* emblica L. | Fruit | ChP |
|  |  | Maidong | 麦冬 | *Ophiopogon aponicas* (Thunb.) Ker-Gawl. | Root tuber | ChP |
|  |  | Dihuang | 地黄 | *Rehmannia glutinosa* Libosch. | Root tuber | ChP |
|  |  | Chisao* | 赤芍 | *Paeonia lactiflora* Pall | Root | ChP |
|  |  | Gancao* | 甘草 | *Glycyrrhiza uralensis* Fisch. | Rhizome and root | ChP |
| 62 | Yun Wei Ning Capsule (YWNC) | Yanbaicai | 岩白菜 | *Bergenia purpurascens* (Hook.f.et Thoms.) Engl. var. delavayi (Franch.) Engl. et Irm. | Rhizome | ChP |
|  |  | Mantuoluoye | 曼陀罗叶 | *Datura stramonium* L. | Leaf | SYNP |
| 63 | Zhi Xuan An Shen Granular (ZXASG) | Luxiancao* | 鹿衔草 | *Pyrola calliantha* H. Andres. | Whole plant | ChP |
|  |  | Yinyanghuo * | 淫羊藿 | *Epimedium brevicornum* Maxim. | Leaf | ChP |
|  |  | Huangqi* | 黄芪 | *Astragalus membranaceus* (Fisch.) Bge. Var. mongholicus (Bge.) Hsiao | Root | ChP |
|  |  | Danggui | 当归 | *Angelica sinensis* (Oliv.) Diels | Root | ChP |
|  |  | Chuanxiong | 川芎 | *Ligusticum chuanxiong* Hort. | Rhizome | ChP |
|  |  | Gegen | 葛根 | *Pueraria lobata* (Willd.) Ohwi | Root | ChP |
|  |  | Banxia | 半夏 | *Pinellia aponic* (Thunb.) Breit. | Tuber | ChP |
|  |  | Gancao* | 甘草 | *Glycyrrhiza uralensis* Fisch. | Rhizome and root | ChP |
|  |  | Suanzaoren | 酸枣仁 | *Ziziphus aponi* Mill. Var. spinosa (Bunge) Hu ex H.F. Chou | Seed | ChP |
|  |  | Ganjiang | 干姜 | *Zingiber officinale* Rosc. | Rhizome | ChP |
|  |  | Zexie | 泽泻 | *Alisma orientalis* (Sam.) Juzep. | Tuber | ChP |
|  |  | Baizhu | 白术 | *Atractylodes macrocephala* Koidz. | Rhizome | ChP |
| 64 | Zhong Tong Liniment (ZTL) | Sanqi | 三七 | *Panax notoginseng* (Burk.) F. H. Chen | Rhizome and root | ChP |
|  |  | Jintiesuo | 金铁锁 | *Psammosilene tunicoides* W. C. Wu et C. Y. Wu | Root | ChP |
|  |  | Chonglou* | 重楼 | *Paris polyphylla* Smith var. chinenisi (Franch) Hara | Rhizome | ChP |
|  |  | Dengzhanxixin | 灯盏细辛 | *Erigeron breviscapus* (Vaniot) Hand. -Mazz. | Whole plant | ChP |
|  |  | Baiji | 白及 | *Bletilla striata* (Thunb.) Reichb.f. | Tuber | ChP |
|  |  | Bohe | 薄荷脑 | DL-Menthol | - | ChP |
|  |  | Gancao* | 甘草 | *Glycyrrhiza uralensis* Fisch. | Rhizome and root | ChP |
|  |  | Bingpian | 冰片 | C_10_H_18_O | Mineral | ChP |
|  |  | Shexiang* | 麝香 | *Moschus berezovskii* Flerov | Secretion | ChP |
|  |  | Zhizi | 栀子 | *Gardenia jasminoides* Ellis | Fruit | ChP |
|  |  | Baizhi* | 白芷 | *Angelica dahurica* (Fisch.ex Hoffm.) Benth. Et Hook.f. | Root | ChP |
|  |  | Huobahuagen | 火把花根 | *Tripterygium hypoglaucum* (Levl.) Hutch | Root | SYNP |
|  |  | Yuputaogen | 玉葡萄根 | *Amoelopsis delavayana* (Franch.) Planch. | Root | SYNP |
|  |  | Qiyelian | 七叶莲 | *Schefflera venulosa* (Wight et Arn.) Harms | Whole plant | SYNP |
|  |  | Jinyezi | 金叶子 | *Craibiodendron yunnanense* W.W. Smith | Leaf | SYNP |
|  |  | Bajiaolian | 八角莲 | *Dysosma versipellis* (Hance) M. Cheng ex Ying | Rhizome | SYNP |
|  |  | Xueshangyizhihao* | 雪上一枝蒿 | *Aconitum brachypodum* Diels | Root tuber | SHNP |
|  |  | Pimacao | 披麻草 | *Veratrum mengzeanum* Loes.f. | Root | SFJP |
|  |  | Diancaowu | 滇草乌 | Unknown | - | NO |
| 65 | Zidan Huo Xue Tablet (ZHXT) | Total saponins of Sanqi | 三七总皂苷 | *Panax notoginseng* (Burk.) F. H. Chen | Extract | ChP |
|  |  | Zidanshen | 紫丹参 | *Salvia yunnanensis* C. H. Wright | Root | SYNP |
| 66 | Zi Deng Capsule (ZDC) | Dengzhanxixin | 灯盏细辛 | *Erigeron breviscapus* (Vaniot) Hand. -Mazz. | Whole plant | ChP |
|  |  | Sanqi | 三七 | *Panax notoginseng* (Burk.) F. H. Chen | Rhizome and root | ChP |
|  |  | Gegen | 葛根 | *Pueraria lobata* (Willd.) Ohwi | Root | ChP |
|  |  | Gancao* | 甘草 | *Glycyrrhiza uralensis* Fisch. | Rhizome and root | ChP |
|  |  | Zidanshen | 紫丹参 | *Salvia yunnanensis* C.H. Wright | Root | SYNP |
| 67 | Zi Jiao Xuan Tincture (ZJXT) | Gonglaomu* | 功劳木 | *Mahonia bealei* (Fort.) Carr. | Stem | ChP |
|  |  | Kushen | 苦参 | *Sophora flavescens* Ait. | Root | ChP |
|  |  | Zihuadiding | 紫花地丁 | *Viola yedoensis* Makino | Whole plant | ChP |
|  |  | Huajiao* | 花椒 | *Zanthoxylum schinifolium* Sieb.et Zucc. | Fruit peel | ChP |
|  |  | Wuweizi* | 五倍子 | *Rhus potaninii* Maxim | Gall | ChP |
| 68 | Fufang Luxiancao Capsule (FFLC) | Tufuling | 土茯苓 | *Smilax glabra* Roxb. | Rhizome | ChP |
|  |  | Kushen | 苦参 | *Sophora flavescens* Ait. | Root | ChP |
|  |  | Tianhuafen | 天花粉 | *Trichosanthes kirilowii* Maxim | Root | ChP |
|  |  | Jiuxiangchong | 九香虫 | *Aspongopus chinensis* Dallas | Insect body | ChP |
|  |  | Luxiancao | 鹿仙草 | *Balanophora harlandii* Hook.f. | Whole plant | SYNP |
|  |  | Huangyaozi | 黄药子 | *Dioscorea bulbifera* L. | Tuber | SGDP |
| 69 | Hong Jin Xiao Jie Pill (HJXJP) | Sanqi | 三七 | *Panax notoginseng* (Burk.) F. H. Chen | Rhizome and root | ChP |
|  |  | Xiangfu | 香附 | *Cyperus rotundus* L. | Rhizome | ChP |
|  |  | Chaihu* | 柴胡 | *Bupleurum chinense* DC. | Root | ChP |
|  |  | Bajiaolian | 八角莲 | *Dysosma versipellis* (Hance) M. Cheng ex Ying | Rhizome | SYNP |
|  |  | Wuxiangxueteng | 五香血藤 | *Kadsura longipedunculata* Finet et Gagnep. | Cane | SYNP |
|  |  | Heimayi | 黑蚂蚁 | *Polyrhachis dives* Smith | Insect body | SYNP |
|  |  | Dahongpao | 大红袍 | *Campylotropis hirtella* (Franchet) Schindler | Root | SHNP |
|  |  | Jishiteng | 鸡矢藤 | *Paederia scandens* (Lour.) Merr. | Whole plant | SHNP |
|  |  | Jinqiaomai | 金荞麦 | *Fagopyrum dibotrys* (D. Don) Hara | Rhizome | ChP |
|  |  | Shufuchong* | 鼠妇虫 | *Armadillidium vulgare* Latreille | Insect body | SSDP |
| 70 | Hong Jin Xiao Jie Capsule (HJXJC) | Sanqi | 三七 | *Panax notoginseng* (Burk.) F. H. Chen | Rhizome and root | ChP |
|  |  | Xiangfu | 香附 | *Cyperus rotundus* L. | Rhizome | ChP |
|  |  | Chaihu* | 柴胡 | *Bupleurum chinese* DC. | Root | ChP |
|  |  | Jinqiaomai | 金荞麦 | *Fagopyrum dibotrys* (D. Don) Hara | Rhizome | ChP |
|  |  | Wuxiangxueteng | 五香血藤 | *Kadsura longipedunculata* Finet et Gagnep. | Cane | SYNP |
|  |  | Dahongpao | 大红袍 | *Campylotropis hirtella* (Franchet) Schindler | Root | SHNP |
|  |  | Heimayi | 黑蚂蚁 | *Polyrhachis dives* Smith | Insect body | SYNP |
|  |  | Bajiaolian | 八角莲 | *Dysosma versipellis* (Hance) M. Cheng ex Ying | Rhizome | SYNP |
|  |  | Shufuchong* | 鼠妇虫 | *Armadillidium vulgare* Latreille | Insect body | SSDP |
|  |  | Jishiteng | 鸡矢藤 | *Paederia scandens* (Lour.) Merr. | Whole plant | SHNP |
| 71 | Shu Lie An Capsule (SLAC) | Dafabiao | 大发表 | *Campylotropis trigonoclada* (Franch.) A. K. Schindl. | Whole plant | SYNP |
| 72 | Wu Jin Huoxue Zhitong Tablet (WJHXZTT) | Sanqi | 三七 | *Panax notoginseng* (Burk.) F. H. Chen | Rhizome and root | ChP |
|  |  | Xiangfu | 香附 | *Cyperus rotundus* L. | Rhizome | ChP |
|  |  | Chaihu* | 柴胡 | *Bupleurum chinese* DC. | Root | ChP |
|  |  | Jinqiaomai | 金荞麦 | *Fagopyrum dibotrys* (D. Don) Hara | Rhizome | ChP |
|  |  | Wuxiangxueteng | 五香血藤 | *Kadsura longipedunculata* Finet et Gagnep. | Cane | SYNP |
|  |  | Dahongpao | 大红袍 | *Campylotropis hirtella* (Franchet) Schindler | Root | SHNP |
|  |  | Heimayi | 黑蚂蚁 | *Polyrhachis dives* Smith | Insect body | SYNP |
|  |  | Bajiaolian | 八角莲 | *Dysosma versipellis* (Hance) M. Cheng ex Ying | Rhizome | SYNP |
|  |  | Shufuchong* | 鼠妇虫 | *Armadillidium vulgare* Latreille | Insect body | SSDP |
|  |  | Jishiteng | 鸡矢藤 | *Paederia scandens* (Lour.) Merr. | Whole plant | SHNP |
| 73 | Jin Wei Tai Capsule (JWTC) | Huanglian | 黄连 | *Coptis chinensis* Franch | Rhizome | ChP |
|  |  | Sharen* | 砂仁 | *Amomum villosum* Lour. | Fruit | ChP |
|  |  | Yanhusuo | 延胡索 | *Corydalis yanhusuo* W. T. Wang | Tuber | ChP |
|  |  | Muxiang | 木香 | *Aucklandia lappa* Decne. | Root | ChP |
|  |  | Jinqiaomai | 金荞麦 | *Fagopyrum dibotrys* (D. Don) Hara | Rhizome | ChP |
|  |  | Dahongpao | 大红袍 | *Campylotropis hirtella* (Franchet) Schindler | Root | SHNP |
|  |  | Guanzhong | 管仲 | *Potentilla fulgens* Wall.ex Hook | Root | SYNP |
|  |  | Jishiteng | 鸡矢藤 | *Paederia scandens* (Lour.) Merr. | Whole plant | SHNP |

Note: * means that medicine has more origins of species. YPM: Yi patent medicine; Ref. : Reference; ChP: Chinese Pharmacopoeia; SYNP: Standards for Chinese medicinal materials in Yunnan Province; SSDP: Standards for Chinese medicinal materials in Shandong Province (2012); SSCP: Standards for Chinese medicinal materials in Sichuan Province (2010); SGZP: Standards for Chinese medicinal materials in Guizhou Province (2009); SHNP: Standards for Chinese medicinal materials in Hunan Province (2010); SGDP: Standards for Chinese medicinal materials in Guangdong Province (2011); SHLP: Standards for Chinese medicinal materials in Heilongjiang Province (2001); SFJP: Standards for Chinese medicinal materials in Fujian Province (2006); SSXP: Standards for Chinese medicinal materials in Shaanxi Province (2015); SSHP: Standards for Chinese medicinal materials in Shanghai (1994)
